# Lytic bacteriophage disrupts biofilm and inhibits growth of pan-drug-resistant *Listeria monocytogenes* in dairy products

**DOI:** 10.3389/fmicb.2025.1653368

**Published:** 2025-08-04

**Authors:** Elsayed M. Abdullah, Eman Y. T. Elariny, Rewan Abdelaziz, Abeer S. Albalawi, Abeer M. Almutrafy, Mohamed Samir A. Zaki, Safaa A. Abdel-Karim, Yasmine H. Tartor

**Affiliations:** ^1^Department of Microbiology and Botany, Faculty of Science, Zagazig University, Zagazig, Egypt; ^2^Department of Microbiology, Faculty of Science, Ain Shams University, Cairo, Egypt; ^3^Department of Biology, College of Science, Taibah University, Madinah, Saudi Arabia; ^4^Department of Anatomy, College of Medicine, King Khalid University, Abha, Saudi Arabia; ^5^Department of Microbiology and Immunology, Faculty of Pharmacy, Zagazig University, Zagazig, Egypt; ^6^Department of Microbiology, Faculty of Veterinary Medicine, Zagazig University, Zagazig, Egypt

**Keywords:** biocontrol, bacteriophage, foodborne pathogens, anti-biofilm agents, milk, cheese, food safety, *L. monocytogenes*

## Abstract

*Listeria monocytogenes* is a major foodborne pathogen whose presence presents a continuous challenge in the food industry. A key issue is the formation of biofilms, which are complex microbial communities that cling to surfaces. These biofilms are incredibly resilient, making them tough to eliminate and manage. Therefore, it is crucial to find new and innovative ways to prevent and remove them. This study investigated the prevalence of *L. monocytogenes* in raw milk and Kareish cheese samples, as well as its resistance to antimicrobials and its ability to form biofilms. We also isolated and characterized a lytic bacteriophage to explore its anti-biofilm potential. *Listeria* species prevalence was 20% (*n* = 24/120 samples), higher in raw milk (31.7%) than Kareish cheese (8.3%). Eighteen isolates (15%) were identified as *L. monocytogenes*. High resistance rates were observed, notably to cefotaxime and cotrimoxazole. One pan-drug resistant (PDR) isolate was found in Kareish cheese, and the other 17 isolates were multidrug resistant (MDR). All *L. monocytogenes* isolates formed biofilms, categorized as weak: *n* = 7, moderate: *n* = 9, and strong: *n* = 2. We isolated a lytic bacteriophage, vB_LmoP_M15, which demonstrated lytic activity against all *L. monocytogenes* isolates, including both MDR and PDR strains. This phage belongs to the *Podoviridae* family, characterized by a short, non-contractile tail and an icosahedral head. Its genome size was estimated to be approximately 48.5 kb based on agarose gel electrophoresis of undigested phage DNA using a high molecular weight marker, and its restriction pattern was analyzed using *Hin*fI*, Hin*dIII, and *Hae*III enzymes. It has a latent period of 15 min and a burst size of 172 phage particles per infected cell. Phage vB_LmoP_M15 demonstrated significant antibiofilm activity (*p* < 0.05 to *p* < 0.0001). It effectively disrupted preformed biofilms and inhibited biofilm formation by MDR/PDR isolates. Application of vB_LmoP_M15 in pasteurized milk resulted in a significant reduction of *L. monocytogenes* counts by 2.45 log_10_ CFU/ml over 7 days at 30°C. These findings underscore the significant potential of phage vB_LmoP_M15 for controlling *L. monocytogenes* contamination and biofilms in dairy products.

## 1 Introduction

*Listeria monocytogenes* is a Gram-positive, facultative anaerobic bacterium that represents a considerable risk to public health as a prominent foodborne pathogen. It is responsible for listeriosis, a serious infection that predominantly impacts individuals with weakened immune systems, including pregnant women, newborns, and older adults. Listeriosis has the potential to result in severe health complications, including meningitis, septicemia, and spontaneous abortions, with a mortality rate that can reach as high as 30% among vulnerable groups (Swaminathan and Gerner-Smidt, 2007). The capacity of *L. monocytogenes* to flourish in extreme conditions, such as refrigeration temperatures, elevated salinity, and low pH, presents significant challenges for control in food processing and storage settings (Vázquez-Boland et al., 2001).

Dairy products, especially raw milk and soft cheeses, have been frequently implicated in outbreaks of listeriosis worldwide (Gandhi and Chikindas, 2007). In developing countries, where regulatory standards and hygiene practices may be less stringent, the risk of contamination is even higher (Jackson et al., 2018). The persistence of *Listeria* in food products underscores the need for effective detection and control measures to mitigate its impact on public health (Ramaswamy et al., 2007).

Although multidrug-resistant (MDR) strains of *L. monocytogenes* remain relatively rare, their recent emergence has introduced new challenges to the effective management of listeriosis. The widespread use of antibiotics in both clinical and agricultural settings has contributed to the development of antimicrobial resistance (AMR), limiting the efficacy of conventional antibiotic therapies (Morvan et al., 2010). The rising occurrence of antimicrobial resistance (AMR), recognized by the World Health Organization (WHO) as a major global public health concern, highlights the urgent need for alternative approaches to combat resistant pathogens (World Health Organization, 2021). This alarming trend highlights the urgent need for alternative strategies, such as bacteriophage therapy, to combat *Listeria* contamination in the food chain (Sulakvelidze et al., 2001).

Moreover, the capacity of *L. monocytogenes* to develop biofilms—organized assemblies of bacteria surrounded by a protective matrix—greatly aids in its survival within food processing settings and boosts its resistance to sanitizers and antimicrobial substances. Bacteria within biofilms often exhibit increased resistance to antibiotics compared to their free-living counterparts, further complicating treatment and control efforts and adding another layer to the challenge posed by antimicrobial resistance (Liu et al., 2024). Despite continuous efforts to control these contaminants, biofilms are incredibly resilient, making them difficult to eradicate and manage. Therefore, it is crucial to explore new and innovative methods for preventing and removing them.

Bacteriophages, viruses that specifically infect and lyse bacterial cells, have gained attention as promising biocontrol agents due to their specificity, efficacy, and safety (Abedon et al., 2011). Bacteriophage therapy, with its unique ability to bypass antibiotic resistance mechanisms, presents a viable solution to this pressing issue (O'Neill, 2016). Phage therapy offers a sustainable and environmentally friendly approach to reducing *Listeria* contamination in food products. While the risk of promoting antibiotic resistance is generally lower compared to antimicrobial agents, careful genomic assessment is essential, as certain phages have been found to harbor antimicrobial resistance genes (Soni and Nannapaneni, 2010; Colavecchio et al., 2017). However, the successful application of phages requires a thorough understanding of their biological properties, including host range, stability under various environmental conditions, and mechanisms of action (Hagens and Loessner, 2010).

While *L. monocytogenes* is a recognized global food safety concern, particularly in dairy (Gandhi and Chikindas, 2007; Jackson et al., 2018), a comprehensive gap remains regarding studies that concurrently investigate detailed AMR profiles, biofilm formation capabilities, and the potential of environmentally isolated biocontrol agents specifically within the context of dairy products like raw milk and Kareish cheese.

This study investigated: (1) the prevalence of *L. monocytogenes* in raw milk and Kareish cheese samples collected from different regions in Egypt; (2) the AMR profiles and biofilm formation ability of the isolates; (3) the isolation and characterization of a lytic *L. monocytogenes* phage, as well as its ability to inhibit biofilm formation and disrupt preformed biofilms; and (4) the application of the lytic bacteriophage to reduce the growth of AMR *L. monocytogenes* in milk. By addressing these objectives, this research aimed to contribute to the development of effective strategies for reducing AMR *L. monocytogenes* contamination in dairy products, ultimately improving food safety and protecting public health.

## 2 Materials and methods

### 2.1 Sampling

A total of 120 samples including raw milk and Kareish cheese samples (60 of each) were collected from diverse sites across Egypt, including Mashtoul Elsouk and Minya Alkamh (Sharkia Governorate), Shibin El-Qanater (Qalyubia Governorate), and El-Bagour (Menoufia Governorate). The samples were collected using aseptic techniques, promptly transported under refrigeration to the laboratory, for immediate bacteriological examination.

### 2.2 Isolation and identification of *Listeria* species

The isolation and detection of *Listeria* species were performed in accordance with the ISO11290-1 standard method (ISO, 1996). The samples underwent a pre-enrichment process utilizing half Fraser broth (Oxoid, Basingstoke, UK) for a duration of 48 h at 37°C, followed by a 24-h enrichment period in Fraser broth (Oxoid, Basingstoke, UK) at the same temperature. Following the enrichment phase, the cultures were transferred onto Palcam agar and Oxford agar (Oxoid, Basingstoke, UK) and incubated at 37°C for a duration of 24 to 48 h. Colonies suspected to be *Listeria*, exhibiting a black coloration with a surrounding black zone due to esculin hydrolysis, were further characterized through Gram staining and a series of biochemical tests, including oxidase, catalase, CAMP, and motility tests (Gasanov et al., 2005).

### 2.3 Identification and molecular characterization of *Listeria* species

Genomic DNA was extracted from bacterial isolates using the QIAamp DNA Mini Kit (Catalogue no. 51304) in accordance with the manufacturer’s guidelines. The extracted DNA’s quality and concentration were evaluated with a NanoDrop spectrophotometer (Thermo Fisher Scientific, Waltham, MA, USA). The DNA was subsequently used for identification of *Listeria* species via PCR amplification and agarose gel electrophoresis. For the detection of *L. monocytogenes*, species-specific primers targeting the 16S rRNA gene (Forward: 5′-GGA CCGGGGCTA ATA CCG AAT GAT AA-3′; Reverse: 5′-TTC ATG TAG GCG AGT TGC AGC CTA-3′) were used, amplifying a 1,200 bp product (Wiedmann et al., 1993). Additionally, primers specific to other *Listeria* species including *L. innocua* (Forward: 5′-GGAGTTATTAACGAAGATACT-3′; Reverse: 5′-TTCTGCTTTTACTTCTTTAGCA-3′, *L. ivanovii* (Forward: 5′-AAGCTGCAGTTATTCATTCC-3′; Reverse: 5′-ATCTAAGAATTTTTGTTTTAGT-3′), *L. welshimeri* (Forward: 5′-TTCTCGTATTATCGGTTTACCA-3′; Reverse: 5′-GCTTCAAGATAGATTTCTTTCAA-3′, and *L. marthii* (Forward: 5′-AGAATATATTTGGAACAGCATC-3′; Reverse: 5′-GTTCGATTGCACGGATGGAAAG-3′ were used (Kim et al., 2015). The amplification reaction comprised 12.5 μl of Emerald Amp GT PCR Master Mix (Takara, Code No. RR310A), 1 μl of each forward and reverse primers (20 pmol), 6 μl of template DNA, and nuclease-free water to a final volume of 25 μl. The PCR was performed using a Thermal Cycler (Biometra T3 Thermocycler, Göttingen, Germany). The cycling conditions for *L. monocytogenes* included an initial denaturation at 94°C for 3 min, followed by 35 cycles of denaturation at 94°C for 1 min, annealing at 60°C for 2 min, and extension at 72°C for 1.5 min. The annealing temperature for other *Listeria* species was set to 54°C for a duration of 1 min. The PCR products, accompanied by suitable positive and negative controls for each run, underwent analysis through 1% agarose gel electrophoresis. The gel underwent staining with ethidium bromide (Sigma-Aldrich, Germany) and was subsequently visualized under UV light utilizing a gel documentation system (Alpha Innotech, CA, USA).

### 2.4 Antimicrobial susceptibility testing

The antibiotic susceptibility of the recovered isolates was assessed using the Kirby-Bauer disk diffusion method (Bauer et al., 1966). The isolates were tested with a set of 22 antibiotic discs (Oxoid, UK) that are frequently employed in the management of microbial infections (Supplementary Table 1). The diameters of the inhibition zones were carefully measured to the nearest millimeter (mm) using a calibrated ruler. The isolates were classified as susceptible (S), intermediate (I), or resistant (R) to each antibiotic based on the standard reference values set by the Clinical and Laboratory Standards Institute (CLSI) (Clinical and Laboratory Standard Institute, 2023) and the European Committee on Antimicrobial Susceptibility Testing (EUCAST) (Testing, 2024). The evaluation of the multiple antibiotic resistances (MAR) index was calculated to determine the extent of antibiotic resistance. The MAR index reflects the proportion of antibiotics that bacteria have developed resistance to in relation to the total number of antibiotics tested (Krumperman, 1983).

### 2.5 Testing biofilm formation ability of *L. monocytogenes*

The capacity of *L. monocytogenes* isolates to form biofilms was assessed through the microtiter plate assay (Djordjevic et al., 2002). Briefly, each isolate was cultured overnight in Tryptic Soy Broth (TSB) (Himedia, Mumbai, India) at a temperature of 37°C. A 100 μl aliquot of the bacterial suspension (10^7^ CFU/ml) was placed into the wells of a sterile 96-well polystyrene microplate, followed by incubation at 37°C for 48 h to facilitate biofilm formation. Following the incubation period, the plates were gently washed three times using 150 μl of distilled water to eliminate any non-adherent cells. Following a 45-min period of air drying, the plates underwent a dyeing process for an additional 45 min with 150 μl of 1% crystal violet. The plates were rinsed with distilled water to remove any residual stain. To elute the stain from the biofilms, 200 μl of 95% ethanol was added to each well, and then 100 μl of the destained solution was transferred to a new microtiter plate. The optical density (OD) of the destained solution was measured at 595 nm using a microtiter plate reader (ELx808-BioTek Instruments, Inc., Burlington, VT, USA). The isolates were categorized into non-biofilm formers (NBF), weak biofilm formers (WBF), moderate biofilm formers (MBF), and strong biofilm formers (SBF) based on the OD values (Stepanovic et al., 2000). The classification was performed using the optical density cut-off value (OD_C_), defined as the mean OD of the negative control plus three standard deviations (SD). The following criteria were applied: OD ≤ OD_C_ (NBF), OD_C_ < OD ≤ 2 × OD_C_ (WBF), 2 × OD_C_ < OD ≤ 4 × OD_C_ (MBF), and OD > 4 × OD_C_ (SBF).

### 2.6 Phage isolation, purification, and propagation

Bacteriophages specific to *L. monocytogenes* were isolated from manure samples collected from dairy farms, following established protocols with minor modifications (Carlton et al., 2005; Kropinski et al., 2009). Briefly, 10 g of fresh manure were mixed thoroughly in 90 milliliters of sterile saline solution and shaken vigorously for a duration of 15 min. The homogenate was subjected to centrifugation at 10,000 × g for 10 min at 4°C to remove particulate matter, and the supernatant was then filtered through a 0.22 μm membrane filter to ensure the elimination of any contaminating bacteria (Carlton et al., 2005).

To enrich the phage, 10 ml of the filtered supernatant were mixed with 10 ml of double-strength TSB and 1 ml (10^8^ CFU/ml) of an overnight culture of *L. monocytogenes* (KCh5), a pan-drug-resistant (PDR) isolate with strong biofilm formation capacity. The solution was kept at 37°C for 24 h, with gentle agitation at 120 rpm throughout the duration. Notably, *L. monocytogenes* does not express flagella at 37°C, which may have limited the isolation of flagella-targeting phages (Gründling et al., 2004). After incubation, the culture was centrifuged at 10,000 × g for 10 min. The supernatant was then filtered through a 0.22 μm membrane filter to isolate the phage lysate (Carlton et al., 2005).

The presence of phages in the lysate was confirmed using a spot test (Hyman and Abedon, 2009). Briefly, after the combination of 3 ml of melted soft agar (0.7% agar in TSB) with 100 μl of an overnight culture of *L. monocytogenes*, the resulting mixture was applied to tryptic soya agar plates that had already solidified (Himedia, Mumbai, India). Once the soft agar had solidified, 10 μl of the phage lysate was carefully applied to the surface of the agar plate. The plates were allowed to dry before being incubated at 37°C for a duration of 24 h. The presence of distinct areas (plaques) at the specified sites indicated the presence of phages (Hyman and Abedon, 2009).

Phage purification was accomplished using the double-layer agar (DLA) technique (Kropinski et al., 2009). Individual plaques were selected with a sterile pipette tip and resuspended in 1 ml of SM buffer (100 mM NaCl, 8 mM MgSO₄, 50 mM Tris–HCl, pH 7.5). The resuspended phages underwent three rounds of plaque purification to guarantee the isolation of a single phage strain (Kropinski et al., 2009).

The propagation of phages necessitated the addition of 100 μl of the purified phage suspension into 10 ml of an overnight culture of *L. monocytogenes* in TSB. The solution was maintained at 37°C for 6–8 h with gentle agitation at 120 rpm until the bacterial culture was fully lysed. The lysate underwent centrifugation at 10,000 × g for 10 min to eliminate bacterial debris, followed by filtration of the supernatant through a 0.22 μm membrane filter. The phage titer was assessed through the DLA method, and the lysate was preserved at 4°C for short-term applications or at −80°C in SM buffer with 15% glycerol for extended storage (Byun et al., 2022).

### 2.7 Identification of *L. monocytogenes* phage

Transmission electron microscopy (TEM) was used to morphologically identify *L. monocytogenes* phage (vB_LmoP_M15). High-titer phage lysates were prepared as described in section 2.6 and exact drops of the lysate were systematically placed onto 200-mesh copper grids that had a carbon-coated collodion membrane. The grids were subjected to negative staining with a 2% (w/v) aqueous uranyl acetate solution, adjusted to a pH of 4.5. Excess stains were removed, and the grids were allowed to air-dry. The samples were analyzed using TEM (Hitachi H600A, Japan) at 80 kV to determine the morphology and size of the phage particles (Ackermann, 2009).

### 2.8 Extraction of phage genomic DNA and restriction analysis

The genomic DNA of vB_LmoP_M15 was extracted using the DNeasy Blood & Tissue Kit (Qiagen, Cat. No. 69581) in accordance with the protocol outlined by Jakočiūnė and Moodley (Jakočiūnė and Moodley, 2018). Briefly, bacterial DNA and RNA were removed by incubating the lysate with DNase I (1 U/μl) and RNase A (10 mg/ml) for 1.5 h at 37°C (Sambrook and Russell, 2001). The phage capsid was digested using proteinase K (20 mg/ml) at 56°C for a period of 1.5 h. DNA purification process was performed in accordance with the kit’s protocol, and the concentration and quality were assessed using a Nanodrop spectrophotometer (Thermo Fisher Scientific, Waltham, MA, USA). The DNA was quantified by measuring absorbance at 260 nm, while purity was assessed using OD260/280 and OD260/230 ratios. The purified DNA was digested with FastDigest® enzymes (*Hin*fI, *Hin*dIII, and *Hae*III) at 37°C for 15 min. The DNA fragments were then separated through agarose gel electrophoresis (0.6% agarose in Tris/Borate/EDTA buffer) and visualized under UV light using a gel documentation system. The aim of the restriction digestion was to obtain a preliminary estimation of the genome size of phage vB_LmoP_M15 based on the restriction patterns.

### 2.9 Evaluation of thermal stability of *L. monocytogenes* phage

The thermal stability of vB_LmoP_M15 was investigated to evaluate their survival under a range of temperature conditions as previously described (Mahmoud et al., 2018). Phage suspensions with an initial titer of 10^9^ PFU/ml were exposed to temperatures varying from 30 to 100°C in a water bath incubator. The duration of each temperature treatment was consistently maintained for 10 min. After incubation, the samples were promptly cooled using running tap water to ensure the stability of the phage particles. The confirmation of phage viability was achieved through the DLA method.

### 2.10 Effect of pH values on *L. monocytogenes* phage

The pH stability of vB_LmoP_M15 was assessed using a phage lysate with an initial concentration of 9.2 × 10^9^ PFU/ml. The phages were combined in a sequence of tubes filled with SM buffer, which was modified to various pH levels (ranging from pH 2 to 13) through the addition of sodium hydroxide or hydrochloric acid. The tubes were subsequently incubated at 30°C for a duration of 24 h. Following the incubation period, the count of surviving phages was assessed through the plaque assay technique (Jamalludeen et al., 2007).

### 2.11 Effect of UV irradiation on the survival of vB_LmoP_M15 phage

The sensitivity of vB_LmoP_M15 phage isolates to UV irradiation was evaluated by exposing 10 ml of phage suspension in sterile Petri plates to UV light at two distinct distances (15 cm and 30 cm) from the UV source. A 30-Watt UV lamp functioning at a wavelength of 254 nm was utilized as the UV source. All experimental procedures were conducted in the absence of light to prevent photoreactivation (Lee and Sobsey, 2011). Phage suspensions were subjected to UV irradiation for various time intervals (0, 20, 40, 60, 80, 100, and 120 min). Following exposure, the infectivity of the phages was assessed utilizing the standard plaque assay method (Nuanualsuwan et al., 2008).

### 2.12 *L. monocytogenes* phage host range

The host range (biological activity) of the phage was determined using the spot test, as previously described (Thiemann et al., 1964; Viazis et al., 2011). The isolated vB_LmoP_M15 with the highest titer was subjected to spot testing against several clinical strains that were recovered previously from milk samples, including *Pseudomonas aeruginosa*, *Klebsiella pneumoniae*, *Staphylococcus aureus*, *Escherichia coli*, *L. monocytogenes*, and *Streptococcus agalctiae*. Twenty microliters of the phage suspension (10^5^ PFU/ml) were inoculated on agar plates previously inoculated with each bacterial strain. Bacterial suspensions were adjusted to 10^8^ CFU/ml prior to spotting. The plates underwent incubation at 37°C for a period of 24–48 h to assess the presence of clear plaques. The presence of a clear lytic zone at the designated site indicated the sensitivity to vB_LmoP_M15. For strains that showed lytic zones in the spot test, phage titers were further quantified using the standard double-layer agar plaque assay and expressed as PFU/ml.

### 2.13 One-step growth curve of *L. monocytogenes* phage

The one-step growth curve of vB_LmoP_M15 phage was established using a standard protocol with slight modifications (Hyman and Abedon, 2009). A culture of *L. monocytogenes* was incubated in TSB at 37°C for a duration of 18 h. The bacterial culture was subsequently exposed to the vB_LmoP_M15 at a multiplicity of infection (MOI) of 0.1. Following a 5-min incubation at 37°C to facilitate phage adsorption to the bacterial cells, phage titration was assessed using the double-layer agar plate method and quantified as PFU/ml. This indicates the preliminary count of phages. Samples were collected at consistent intervals of 5 min over a total period of 70 min. Then, each sample was diluted and then plated onto agar plates to evaluate the number of infective phages at each specified time point. This facilitated the progression of the growth curve. The latency period was determined as the time between phage adsorption and the first observable increase in PFU, indicating the beginning of phage progeny release. The burst size was established by evaluating the number of new phages released from each infected bacterium, necessitating a comparison between the phage count at the peak of the growth curve and the initial phage count. The burst size indicates the mean quantity of phages that are expelled from every infected bacterium. The experiment was conducted on three distinct occasions to guarantee the reliability of the results, and the average values, along with their standard deviations, were documented.

### 2.14 Adsorption rate of *L. monocytogenes* phage

The adsorption rate of vB_LmoP_M15 was assessed using the method outlined by Karumidze et al. (2013). In brief, the filtered lysate of vB_LmoP_M15 was introduced to a log-phase culture of *L. monocytogenes*, serving as an indicator strain, at a multiplicity of infection of 0.1 PFU/CFU/ml. At 1-min intervals, samples of 1 ml were collected for the initial 5 min and subsequently filtered using membrane filters (0.45 μm, Millipore). The filtrate was analyzed through the DLA technique to ascertain the titer of non-adsorbed phages. The rate constant for adsorption (K) was calculated using the following equation: $K=(2.3/(B\times T))\times \log(P_{0}/P)$ , where K is the adsorption rate constant, B is the bacterial host concentration (10^8^ CFU/ml), T is the time (min), P₀ is the unadsorbed phage concentration at the beginning, and P is the unadsorbed phage concentration at the end.

### 2.15 Antibiofilm potential of *L. monocytogenes* phage

#### 2.15.1 Disruption of preformed biofilm

To evaluate the capacity of phage to disrupt the established biofilms, biofilms were initially formed as outlined in the preceding section. Following a 48-h incubation period, the culture medium was carefully aspirated, and the wells were washed twice with 200 μl of phosphate-buffered saline (PBS) to eliminate loosely attached cells. Following this, 200 μl of phage solution (10^9^ PFU/ml) was introduced to one set of wells, whereas a separate group was treated with 200 μl of sterile saline as a control. The plates were incubated at 37°C for a further duration of 24 h. After the incubation period, the wells were washed twice with sterile saline and were subsequently stained using a 1% crystal violet solution. The OD was measured at 595 nm, and the decrease in biofilm biomass was evaluated by comparing the OD values of the phage-treated wells with those of the control wells (Migliaccio et al., 2024).

#### 2.15.2 Inhibition of biofilm formation

The ability of phage to prevent biofilm formation was also investigated. At the initiation of the assay, 100 μl of *L. monocytogenes* cell suspension (10^7^ CFU/ml) and 100 μl of phage solution (10^9^ PFU/ml) were mixed in a sterile microtiter plate. The plates were incubated at 37°C for 48 h. Following the incubation period, the wells were washed, stained with crystal violet, and the optical density was assessed at 595 nm as previously described. The assessment of biofilm formation inhibition involved comparing the OD values of wells treated with phage to those of control wells that did not receive phage treatment.

### 2.16 Effect of phage on *L. monocytogenes* growth in pasteurized milk

Pasteurized full-fat milk (3% fat) was obtained from a nearby retail store. Prior to inoculation, the milk was tested to ensure the absence of *L. monocytogenes* and *Listeria*-specific phages. Both were not detected in the tested samples. The experimental protocol used for the application assay was adapted from a previously established method (Stone et al., 2020). An overnight culture of *L. monocytogenes* was prepared, subsequently diluted 1:5 in TSB, and incubated for 3 h at 25°C. Bacterial cells were diluted in maximum recovery diluent (Oxoid Ltd., Basingstoke, UK) to achieve an initial concentration of 3.0 log_10_ CFU/ml in the milk. Calcium chloride (CaCl2, Merck, Germany) was added to the milk at a final concentration of 18.5 mM to enhance phage activity. The vB_LmoP_M15 was propagated and introduced to the test samples at a Multiplicity of Infection (MOI) of 100, while the control samples remained untreated. All samples were incubated at 30°C for 7 days to mimic potential temperature abuse during handling, storage, or retail display. Bacterial viable counts (CFU/ml) were evaluated immediately following the addition of phage (T0) and subsequently at intervals of days 1, 3, 5, and 7. Samples were subjected to serial dilution to enumerate *L. monocytogenes* and then plated onto *Listeria* Chromogenic Agar (Himedia, India). The plates were incubated at 37°C for 48 h.

### 2.17 Data analysis

The analysis of the data was conducted utilizing SPSS version 26 (IBM Corp, Armonk, NY, USA). The chi-square test was employed to examine the variations in the prevalence of *L. monocytogenes* from diverse origins and to evaluate the differences in the AMR patterns of the isolates from various sources. All experimental procedures were conducted in triplicate, and the results were presented as mean ± standard error of the mean (SEM). One-way ANOVA followed by Tukey’s *post hoc* test was used to analyze the phage characterization results, including the latent period, burst size from the one-step growth curve, pH stability, and thermal stability. An independent sample T-test was employed to assess the antibiofilm and antibacterial effectiveness of *L. monocytogenes* phage derived from different sources. The *p*-values were considered statistically significant if they were less than 0.05. Graphs were produced using GraphPad Prism software version 8 (San Diego, CA, USA) and R-software version 4.0.2.^1^

## 3 Results

### 3.1 Isolation and identification of *Listeria* species from dairy products

Out of the 120 samples analyzed (60 raw milk and 60 Kareish cheese), 24 (20%) were positive for *Listeria* species using the ISO11290-1 standard method. Raw milk samples showed a significantly higher prevalence (31.7%, 19/60) compared to Kareish cheese samples (8.3%, 5/60) (*p* = 0.002). Among the positive isolates, *L. monocytogenes* was the most prevalent species, with 18 isolates (15%) identified, while *L. welshimeri* was detected in 6 isolates (5%). The prevalence of *L. welshimeri* was significantly higher in raw milk samples (10%) compared to Kareish cheese samples (0%) (*p* = 0.027). Colonies suspected to be *Listeria* appeared black with a surrounding black zone on selective media due to esculin hydrolysis. The isolates demonstrated characteristics of being Gram-positive rods, were catalase-positive, oxidase-negative, motile at 25°C, and showed distinct hemolysis in the CAMP test. Using species-specific primers targeting the 16S rRNA gene, 18 isolates (15%) were identified as *L. monocytogenes* by a 1,200 bp band, and 6 isolates (5%) as *L. welshimeri* by a 237 bp band. As revealed in Table 1, the prevalence of *Listeria* species varied significantly between raw milk and Kareish cheese samples, with raw milk exhibiting a higher percentage of both *L. monocytogenes* and *L. welshimeri* isolates. To further understand the factors influencing this distribution, a random forest classification analysis revealed that season and location were the most critical predictors for *L. monocytogenes* prevalence.

**Table 1:** Prevalence of *Listeria* species in dairy samples.

Sample type (No.)	Total No. of *Listeria* isolates (%)^a^	No. of *Listeria* species (%)	
		*L. monocytogenes*	*L. welshimeri*
Raw milk (60)	19 (31.7)	13 (21.7)	6 (10)
Kareish cheese (60)	5 (8.3)	5 (8.3)	0
*p*-value	0.002**	0.071	0.027*
Total (120)	24 (20)	18 (15)	6 (5)

*p > 0.05; **p > 0.01.

a The isolation rate was calculated concerning the total number of the examined samples from each source.

### 3.2 Antimicrobial resistance of *Listeria* species

**Figure 1:**
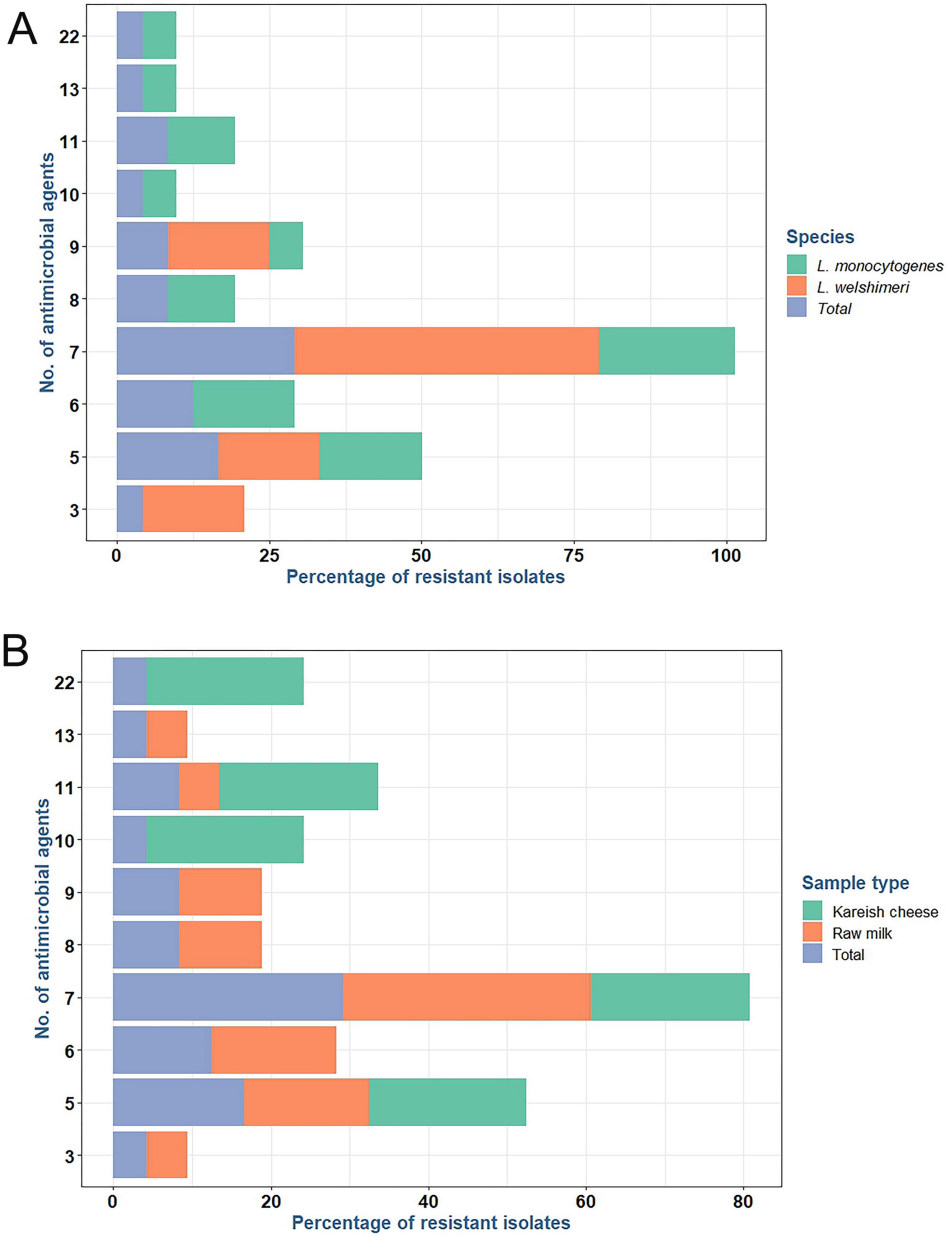
Frequency of resistance to various antimicrobial agents among antimicrobial resistant *L. monocytogenes* and *L. welshimeri* **(A)** recovered from raw milk and kariesh cheese samples **(B)**.

**Figure 2:**
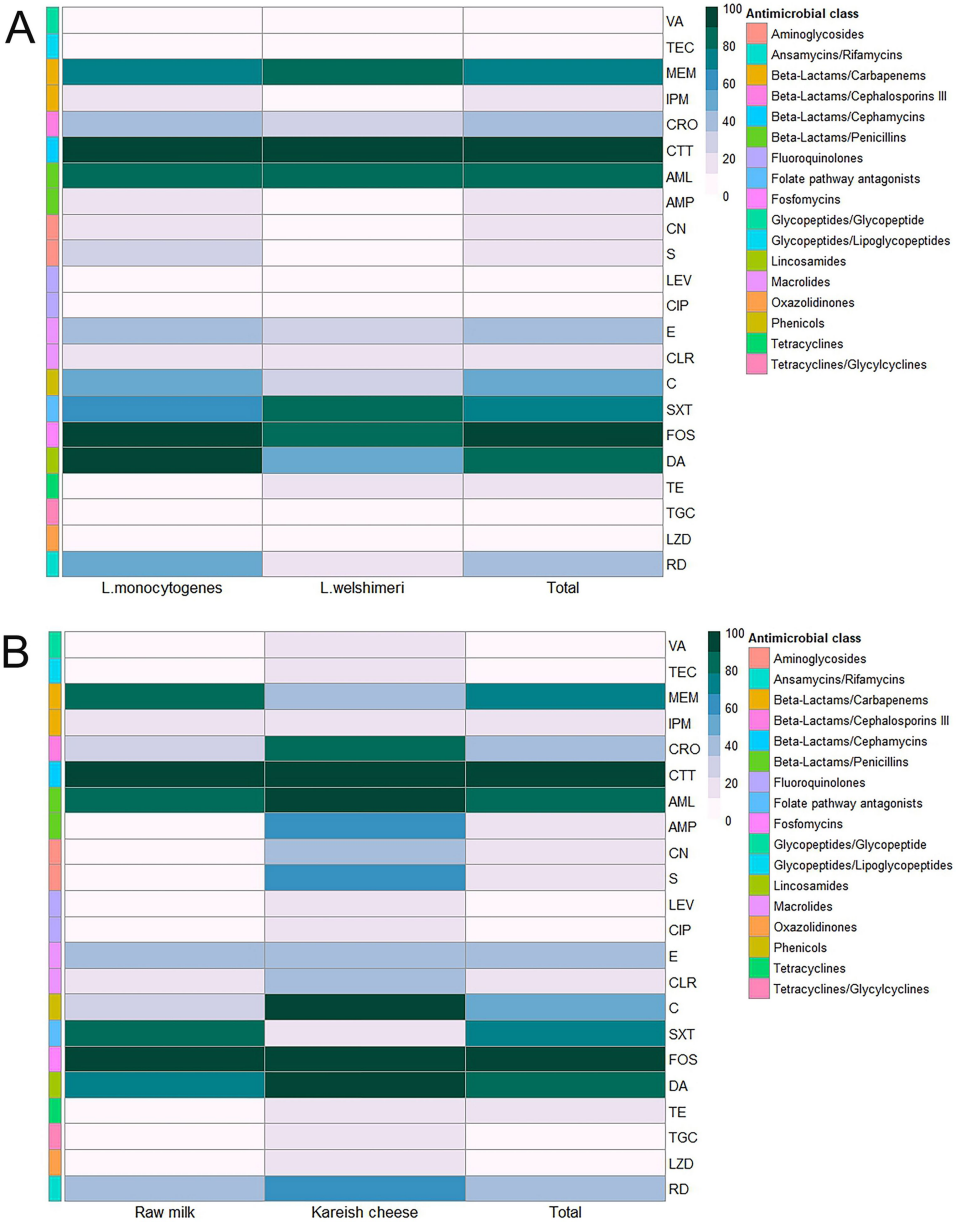
Antimicrobial resistance rates of *Listeria* species **(A)** isolated from raw milk and kariesh cheese samples **(B)**. VA: vancomycin, TEC: teicoplanin, MEM: meropenem, IPM: imipenem, CRO: ceftriaxone, CTT: cefotetan, AML: amoxycillin, AMP: ampicillin, CN: gentamicin, S: streptomycin, LEV: levofloxacin, CIP: ciprofloxacin, E: erythromycin, CLR: clarithromycin, C: chloramphenicol, SXT: trimethoprim/sulfamethoxazole (cotrimoxazole), FOS: fosfomycin, DA: clindamycin, TE: tetracycline, TGC: tigecycline, LZD: linezolid, RD: rifampicin.

*L. monocytogenes* showed a high resistance rate of 95.8% to cotrimoxazole, and *L. welshimeri* isolates were 100% resistant to the same antibiotic. Resistance to ceftriaxone, another cephalosporin, was also prevalent, with 87.5% of *L. monocytogenes* and 83.3% of *L. welshimeri* isolates being resistant. In contrast, all *Listeria* isolates were fully susceptible to vancomycin (VA), teicoplanin (TEC), tigecycline (TGC), levofloxacin (LEV), and ciprofloxacin (CIP), except for the single pan-drug-resistant isolate (KCh5), which showed resistance to all tested antibiotics (Supplementary Table 1). These findings suggest that the mentioned antibiotics remain effective for most *Listeria* infections. Intermediate resistance was observed for erythromycin (45.8% in *L. monocytogenes* and 33.3% in *L. welshimeri*) and rifampicin (20.8% in *L. monocytogenes* and 16.7% in *L. welshimeri*), indicating potential challenges in treatment with these agents. There were statistically significant differences in the resistance rates of *Listeria* species isolates from raw milk and Kareish cheese samples to ampicillin, streptomycin, chloramphenicol, and trimethoprim/sulfamethoxazole (*p* = 0.018, 0.042, 0.011, and 0.028, respectively). Meanwhile, there were no statistically significant differences (*p* > 0.05) in the resistance rates of *Listeria* species isolates from different origins to the other antimicrobials investigated (Supplementary Table 1). Figures 1A,B illustrates the antimicrobial resistance rates of *Listeria* species isolates from different sources, highlighting the varying resistance patterns depending on the source of isolation. Figure 2 complements these findings by showing the frequency of resistance to various antimicrobial agents among *Listeria* isolates from different species (A) and sources (B), providing a detailed overview of the resistance profiles.

### 3.3 Pan-drug resistant *L. monocytogenes* from Kareish cheese

**Figure 3:**
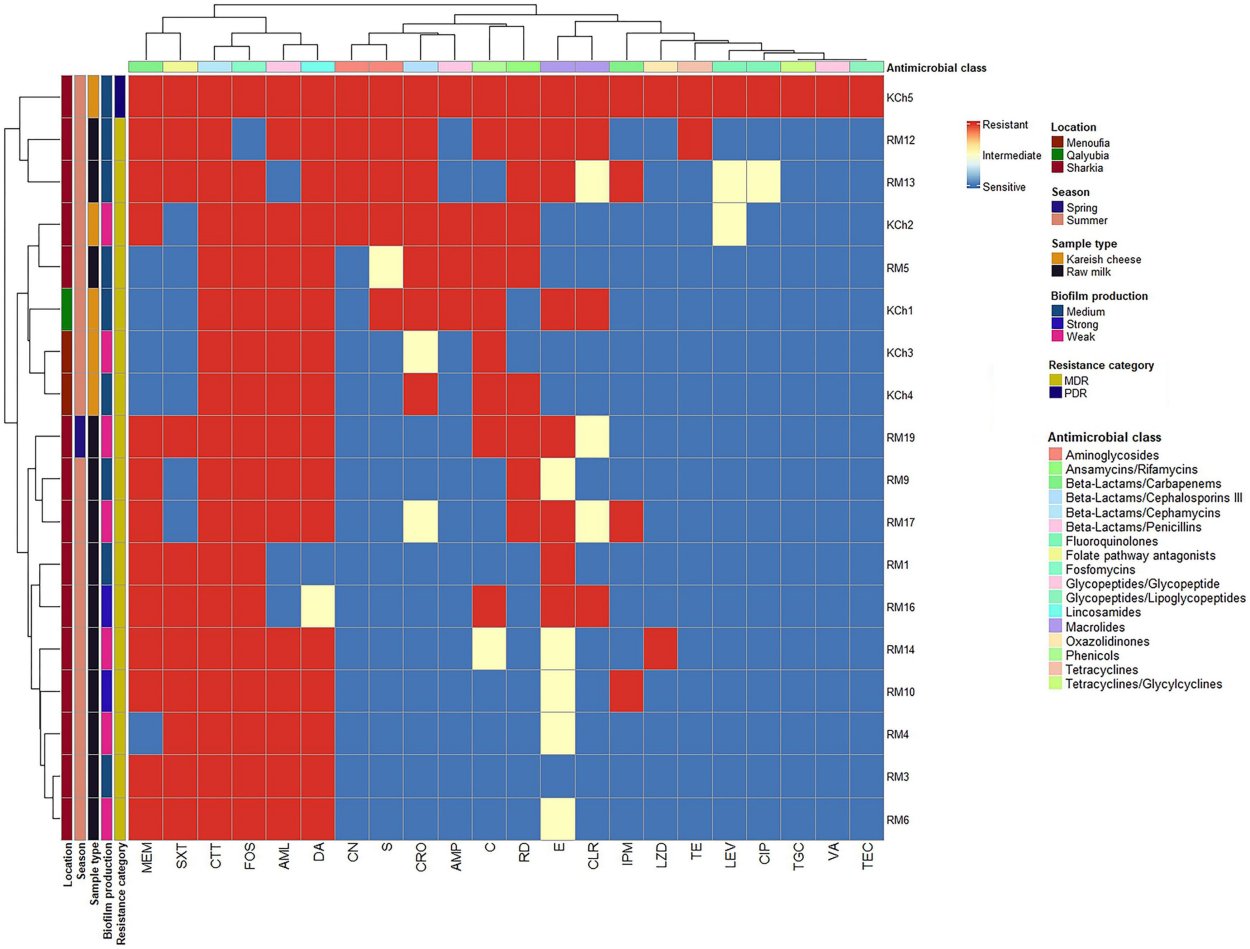
Hierarchical clustering heatmap shows the overall distribution of the 18 investigated *Listeria monocytogenes* isolates based on the antimicrobial resistance pattern and biofilm formation. VA: vancomycin, TEC: teicoplanin, MEM: meropenem, IPM: imipenem, CRO: ceftriaxone, CTT: cefotetan, AML: amoxycillin, AMP: ampicillin, CN: gentamicin, S: streptomycin, LEV: levofloxacin, CIP: ciprofloxacin, E: erythromycin, CLR: clarithromycin, C: chloramphenicol, SXT: trimethoprim/sulfamethoxazole (cotrimoxazole), FOS: fosfomycin, DA: clindamycin, TE: tetracycline, TGC: tigecycline, LZD: linezolid, RD: rifampicin.

Seventeen *L. monocytogenes* isolates (94.4%) exhibited a multidrug-resistant (MDR) phenotype. There were no statistically significant differences (*p* > 0.05) in the MAR index among isolates. However, one *L. monocytogenes* (code no. kch5) isolated from Kareish cheese exhibited complete resistance to all 22 antibiotics tested (MAR index = 1.0), highlighting the emergence of a highly resistant *L. monocytogenes* strain in the food chain. Figure 3 and Supplementary Figure 1 provide further insights through hierarchical clustering and principal component analysis (PCA), respectively, revealing the diversity and polyclonality of *Listeria* isolates. The hierarchical clustering dendrogram (Figure 3; Supplementary Figures 1, 2) shows the relatedness of *Listeria* species isolates based on their antimicrobial resistance profiles. This analysis revealed distinct resistance clusters among isolates from different sources and geographical locations. The PDR isolate (KCh5) from Kareish cheese demonstrated resistance to all antimicrobial classes tested. Several MDR isolates, notably RM12 and RM19 from raw milk, exhibited resistance to multiple antimicrobial classes, particularly *β*-lactams, cephalosporins, and carbapenems. The clustering analysis further indicated geographical associations in resistance patterns, with isolates from identical regions displaying similar resistance profiles.

### 3.4 Biofilm formation ability of *L. monocytogenes* isolates

**Figure 4:**
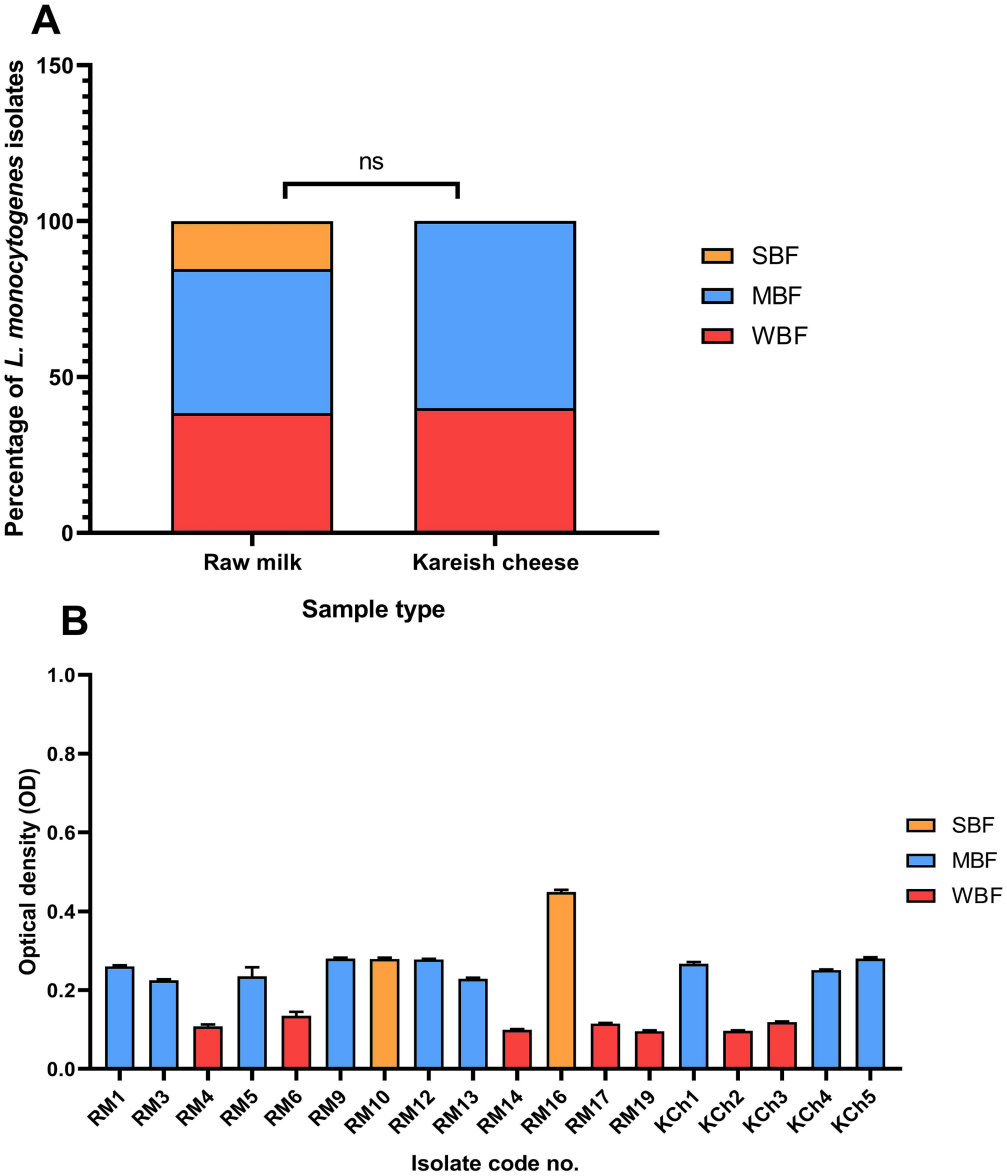
Biofilm formation ability of *Listeria monocytogenes* isolated from raw milk and kareish cheese **(A)** and optical density values at 595 nm **(B)**. SBF: strong biofilm forming, MBF: moderate biofilm forming, WBF: weak biofilm forming, ns, not significant. The code numbers refer to the isolate numbers for raw milk (RM), and kareish cheese (KCh) samples.

The biofilm-forming ability of *L. monocytogenes* isolates obtained from raw milk and kareish cheese samples was quantitatively assessed using a microtiter plate assay. The isolates were classified into three categories based on their OD values at 595 nm: WBF, MBF, and SBF. The analysis revealed that 38.46% (*n* = 5/13) of raw milk isolates were classified as WBF, 46.15% (*n* = 6/13) as MBF, and 15.38% (*n* = 2/13) as SBF. In contrast, 40% (*n* = 2/5) of the kareish cheese isolates were WBF and 60% (*n* = 3/5) were MBF (Figures 4A,B). No significant differences were observed in biofilm-forming capacity between isolates derived from raw milk and kareish cheese (*p* = 0.68).

### 3.5 Phage isolation, purification, and propagation

Using the spot test and DLA technique, a bacteriophage specific to *L. monocytogenes*, designated vB_LmoP_M15, was successfully isolated from manure samples. The phage produced clear plaques on lawns of *L. monocytogenes*, indicating its lytic activity (Supplementary Figure 3). A single plaque was purified and propagated, yielding a high-titer phage lysate. The phage was stored at 4°C for short-term use or at −80°C in SM buffer with 15% glycerol for long-term storage.

### 3.6 Morphotype and genome characterization of *L. monocytogenes* phage

**Figure 5:**
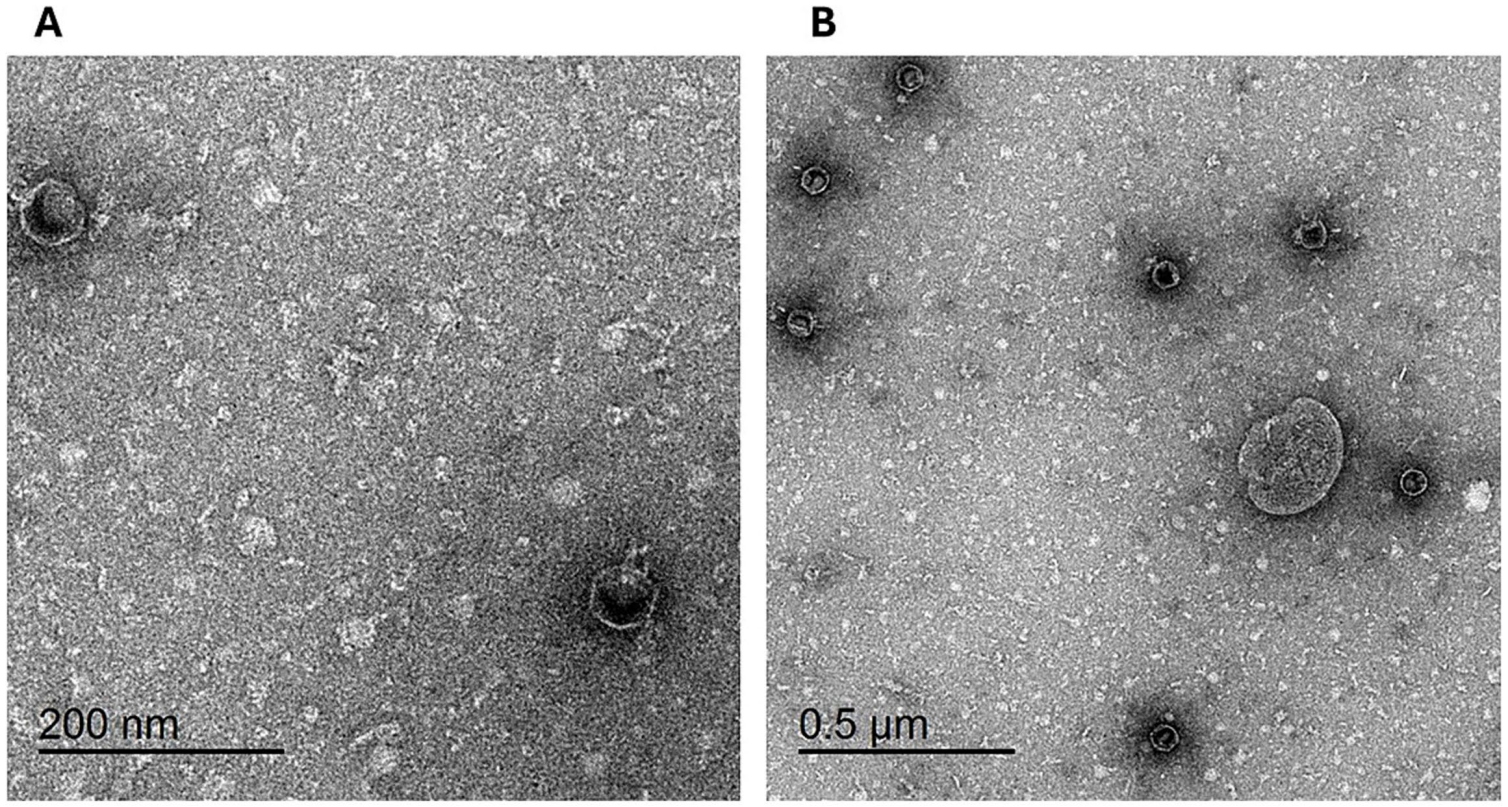
Transmission electron micrograph of phage vB_LmoP_M15: Phage particles with icosahedral heads and short non-contractile tails are visible, consistent with the morphology of the *Podoviridae* family. Scale bars: 200 nm **(A)** and 50 μm **(B)**.

The morphology of the isolated phage was examined using TEM following staining with uranyl acetate. The electron micrographs (Figure 5) reveal that the phage displays icosahedral heads accompanied by short non-contractile tails, a morphology characteristic of phages within the *Podoviridae* family. The phage exhibited head dimensions ranging from 50.42 nm to 56.86 nm, with a notably short tail length, aligning with the distinctive characteristics of the *Podoviridae* family.

The genomic DNA of the vB_LmoP_M15 phage was successfully extracted and analyzed using restriction enzymes. The extracted DNA was visualized on a 0.6% agarose gel, showing a clear band corresponding to the phage genome. Genome size estimation was performed by comparing the undigested DNA band with a high molecular weight marker, suggesting an approximate genome size of 48.5 kb. Restriction fragment length polymorphism (RFLP) analysis was performed using *HinfI*, *HindIII*, and *HaeIII* enzymes. The digestion patterns revealed distinct banding profiles for each enzyme (Supplementary Figure 4).

### 3.7 Effect of temperature on vB_LmoP_M15 survival

**Figure 6:**
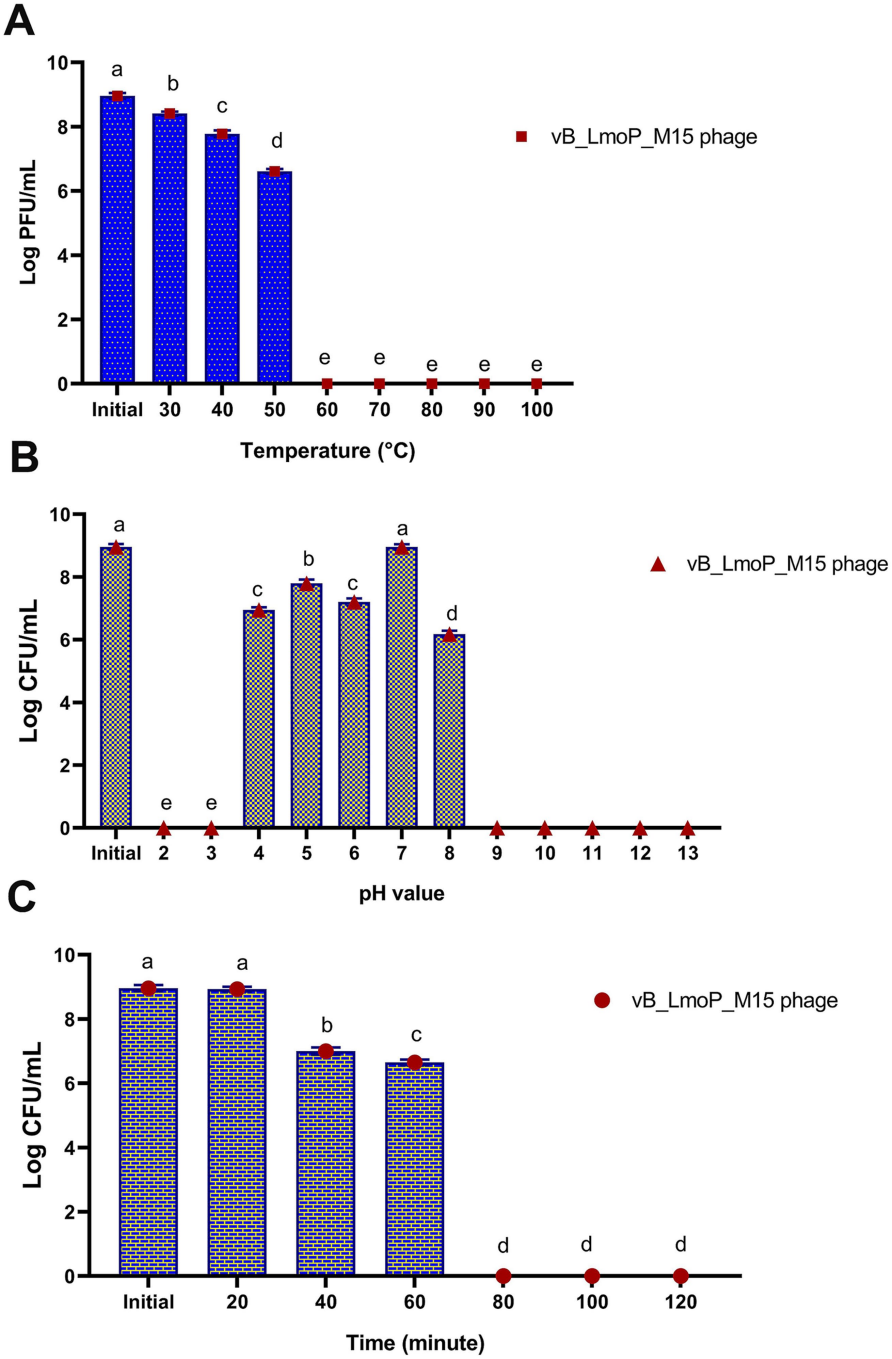
Effect of temperature **(A)**, pH values **(B)**, and U.V. irradiation **(C)** on the survival of vB_LmoP_M15 phage. PFU: plague forming unit; a–e mean values with various superscript letters in the same column vary significantly at *p* < 0.0001.

The thermal stability of vB_LmoP_M15 was assessed at temperatures between 30 and 100°C over a duration of 10 min. The DLA method was employed to assess viability, with the findings presented in Figure 6A. A significant temperature-dependent reduction in phage survival was observed (*p* < 0.0001). At the initial temperature, the phage titer was 8.96 ± 0.092 log PFU/ml. Minimal reduction occurred at 30°C (8.41 ± 0.064 log PFU/ml), but viability declined progressively with increasing temperature. At 40°C, the titer dropped to 7.78 ± 0.103 log PFU/ml (~20% reduction), and at 50°C, it decreased to 6.61 ± 0.075 log PFU/ml (~50% reduction), indicating moderate thermostability. However, at 60°C, the titer was 0 log PFU/ml, and no viable phages were detected at temperatures ≥70°C, demonstrating complete inactivation. These results highlight the temperature sensitivity of vB_LmoP_M15, with significant stability at moderate temperatures (up to 50°C) and complete inactivation at higher temperatures (≥60°C). The thermostability at 50°C, suggests potential applications in moderate-heat environments, while the sensitivity to higher temperatures limits its use in high-heat conditions.

### 3.8 Effect of pH on phage stability

The stability of vB_LmoP_M15 to pH values ranging from 2 to 13 for 24 h at 30°C was examined. The results revealed significant pH-dependent survival (*p* < 0.0001) (Figure 6B). The phage exhibited maximum stability at neutral pH (pH 7) and initial pH, with titers of 8.96 ± 0.087 and 8.96 ± 0.092 log PFU/ml, respectively. Moderate stability was observed at pH 4 (6.95 ± 0.087 log PFU/ml) and pH 5 (7.8 ± 0.116 log PFU/ml), while viability decreased at pH 6 (7.2 ± 0.115 log PFU/ml) and pH 8 (6.18 ± 0.104 log PFU/ml). Extreme pH values (pH 2, 3, and 9–13) resulted in complete inactivation, with no detectable plaques. These findings indicate that vB_LmoP_M15 is highly stable at neutral pH, moderately stable under mildly acidic conditions, and sensitive to extreme pH values. The statistical significance (*p* < 0.0001) underscores the critical role of pH in phage survival, with potential applications in environments with neutral to slightly acidic conditions.

### 3.9 Effect of UV-irradiation on the infectivity of vB_LmoP_M15 phage

The sensitivity of vB_LmoP_M15 phage to UV irradiation (254 nm) for varying durations (0 to 120 min) was evaluated. As presented in Figure 6C, there are significant time-dependent reductions in phage survival (*p* < 0.0001). At 0 min, the phage titer was 8.96 ± 0.098 log PFU/ml, indicating high initial viability. After 20 min, the titer remained stable at 8.93 ± 0.075 log PFU/ml, demonstrating short-term UV resistance. However, viability declined significantly at 40 min (7 ± 0.116 log PFU/ml) and further at 60 min (6.65 ± 0.087 log PFU/ml), indicating moderate UV stability. Prolonged exposure beyond 60 min (80, 100, and 120 min) resulted in complete inactivation, with no detectable plaques. These findings highlight the UV sensitivity of vB_LmoP_M15 phage isolates, with significant stability during short-term exposure (up to 20 min) and moderate stability up to 60 min. Complete inactivation occurred after prolonged exposure, limiting the phage’s utility in high-UV environments.

### 3.10 Adsorption rate and one-step growth curve of vB_LmoP_M15 phage

**Figure 7:**
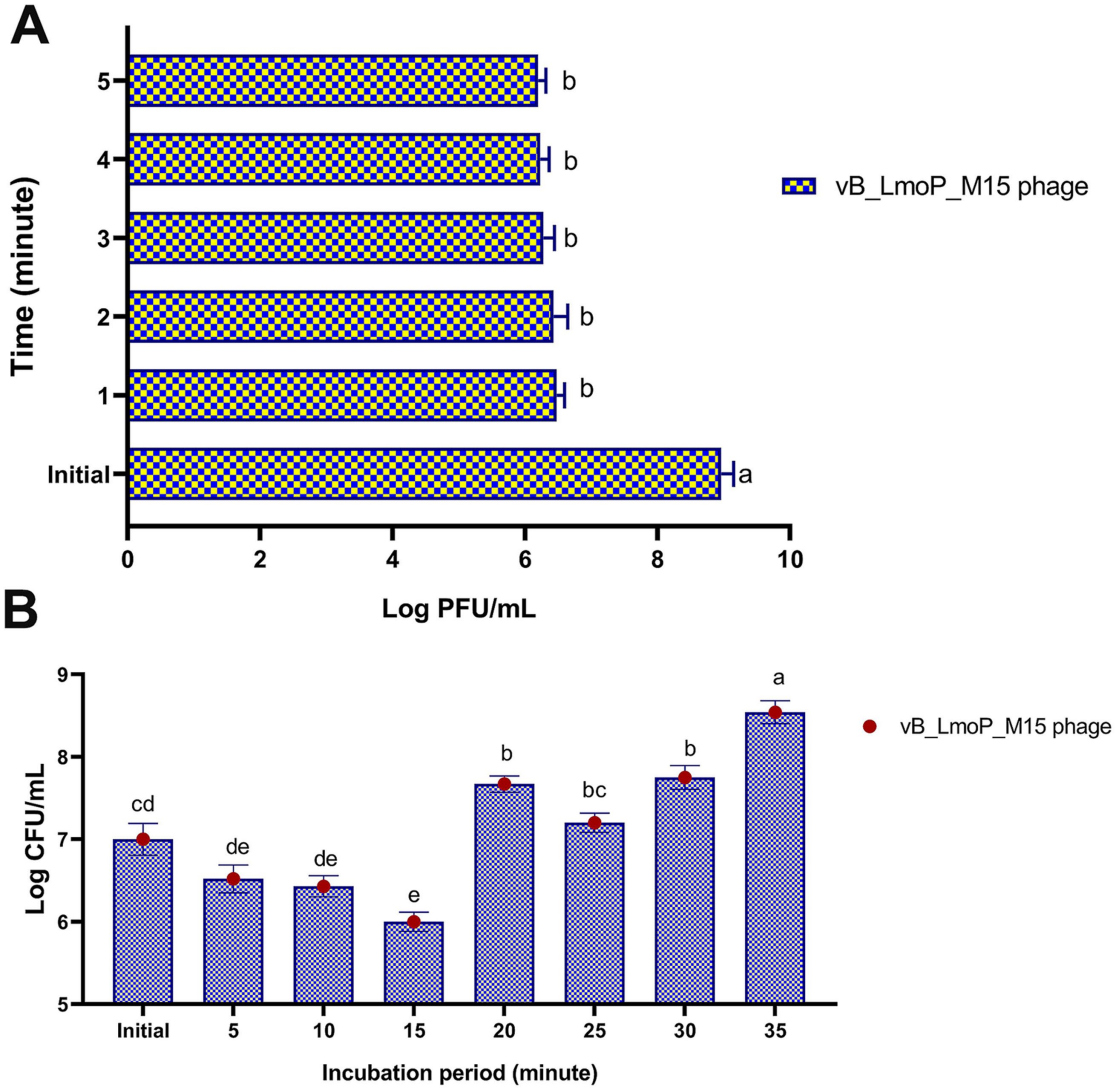
Adsorption rate **(A)** and one-step growth curve **(B)** of vB_LmoP_M15 phage. PFU: plague forming unit; a–e mean values with various superscript letters in the same column vary significantly at *p* < 0.0001.

The adsorption kinetics of vB_LmoP_M15 phage to host cells were evaluated over a 5-min period. Statistically significant differences were observed across the tested time intervals (*p* < 0.0001) (Figure 7A). At the initial time point (0 min), the phage titer was 8.96 ± 0.192 log PFU/ml. Within the first minute, the titer of non-adsorbed phages decreased sharply to 6.48 ± 0.115 log PFU/ml, indicating efficient and rapid attachment to host cells. This trend continued over the subsequent minutes, with titers of 6.43 ± 0.215 log PFU/ml at 2 min, 6.28 ± 0.162 log PFU/ml at 3 min, 6.23 ± 0.133 log PFU/ml at 4 min, and 6.2 ± 0.116 log PFU/ml at 5 min. These findings indicate that the majority of phage adsorption occurred within the first minute, followed by a gradual stabilization in adsorption efficiency. The high adsorption rate observed in this study underscores the efficiency of vB_LmoP_M15 phage in attaching to *L. monocytogenes* host cells, a critical step for initiating the lytic cycle. The results provide significant understanding of the initial phases of phage-host interactions and underscore the promise of vB_LmoP_M15 phage as a potent biocontrol agent targeting *L. monocytogenes*.

The one-step growth curve of vB_LmoP_M15 was determined to elucidate its replication kinetics within *L. monocytogenes* KCh5 host cells. Figure 7B revealed distinct phases of phage infection, including adsorption, eclipse, replication, and release. The initial phage titer was 7 ± 0.192 log PFU/ml (approximately 1 × 10^7^ PFU/ml). During the first 15 min, the titer decreased to 6 ± 0.116 log PFU/ml (approximately 1 × 10⁶ PFU/ml), reflecting the adsorption of phages to host cells and the subsequent eclipse phase, during which no infectious phages are detected. The latent period, characterized as the duration from adsorption to the initiation of progeny release, was 15–20 min. Following the latent period, a sharp increase in phage titer was observed, indicating the onset of the lytic cycle. The titer increases to 7.67 ± 0.098 log PFU/ml at 20 min, 7.2 ± 0.116 log PFU/ml at 25 min, and 7.75 ± 0.144 log PFU/ml at 30 min. The maximum titer reached was 8.54 ± 0.139 log PFU/ml (approximately 3.47 × 10^8^ PFU/ml) at 35 min, indicating the conclusion of the replication cycle and the subsequent release of progeny phages. The burst size, determined by the ratio of the final phage titer to the initial titer, was 172 phages per infected cell. The findings indicate that vB_LmoP_M15 phage shows efficient replication kinetics, characterized by a relatively short latent period and a substantial burst size, underscoring its potential as an effective lytic phage targeting *L. monocytogenes*.

### 3.11 Host range of vB_LmoP_M15 phage

The phage displayed differential lytic activity across the tested strains (Supplementary Table 2). Notably, vB_LmoP_M15 exhibited strong lytic effects (+++) against *L. monocytogenes* and *S. aureus*, with high titers recorded at 9.2 × 10^8^ and 2.2 × 10^7^ PFU/ml, respectively. Moderate lytic activity (++) was observed against certain strains of *S. aureus* and *L. monocytogenes*, with titers ranging between 3.3 × 10^6^ and 9 × 10^6^ PFU/ml. In contrast, weak lysis (+) was detected for *E. coli* and *S. agalactiae*, yielding titers of 1.5 × 10^6^ and 2.7 × 10^6^ PFU/ml, respectively. No lytic activity was observed against *P. aeruginosa*. These titers were determined using plaque assay for strains that showed sensitivity in the spot test, while the spot test was used qualitatively to assess initial lytic potential. Collectively, these findings indicate that the isolated phage possesses a relatively broad host range, with marked efficacy against *L. monocytogenes* and *S. aureus*.

### 3.12 Antibiofilm activity of vB_LmoP_M15 phage

**Figure 8:**
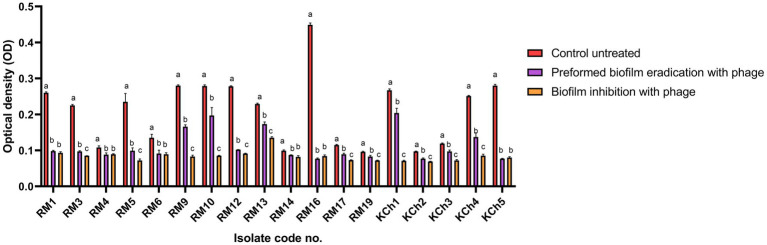
Antibiofilm effect of vB_LmoP_M15 phage on the preformed *Listeria monocytogenes* biofilms and inhibition of biofilm formation. a–c means with various superscript litter within the same column vary significantly at *p* < 0.05.

**Figure 9:**
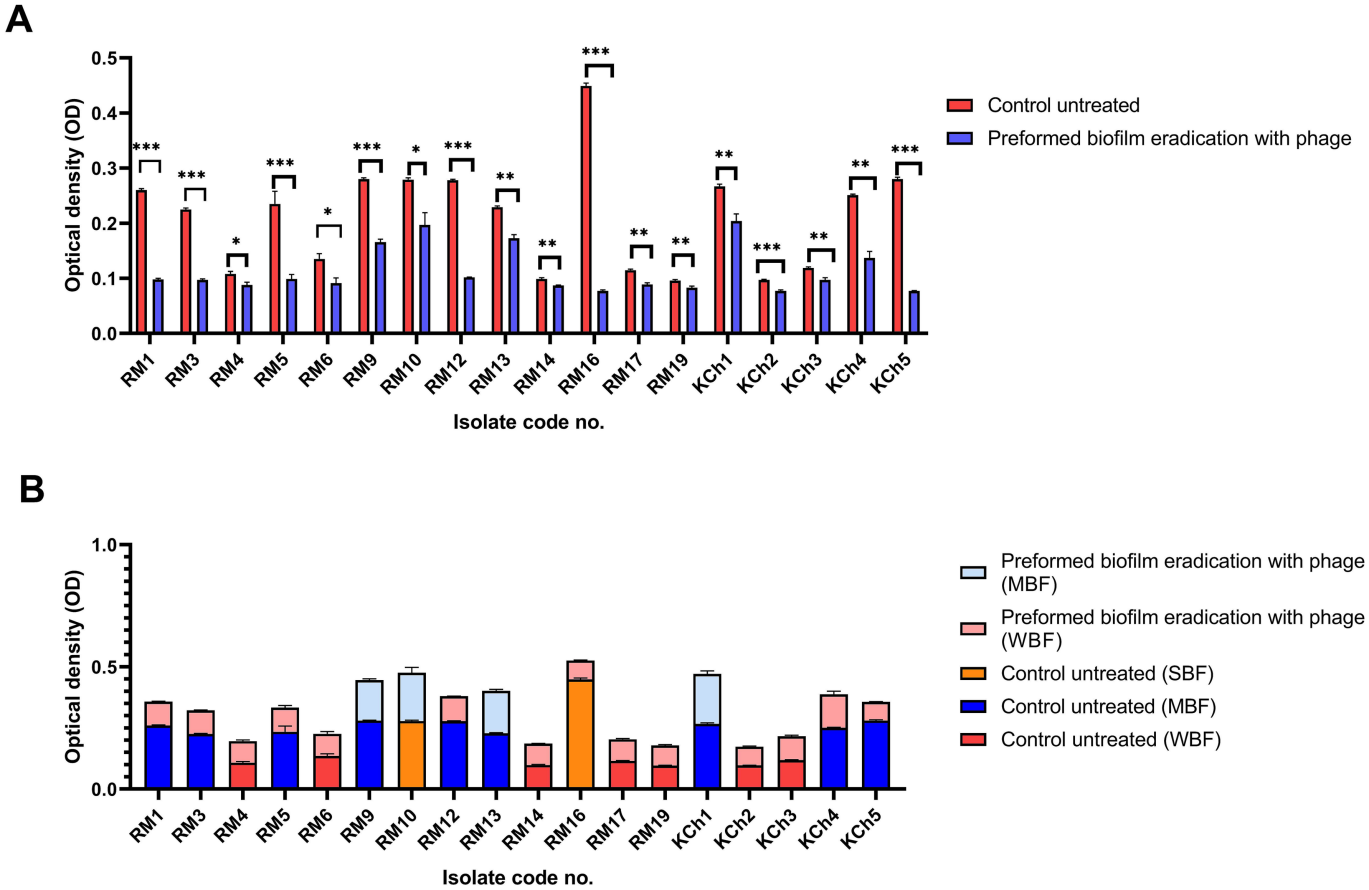
Antibiofilm effect of vB_LmoP_M15 phage on the preformed *Listeria monocytogenes* biofilms **(A)**. Differences in biofilm production abilities among control untreated *L. monocytogenes* isolates and vB_LmoP_M15 phage treated preformed biofilms **(B)**. ns: non-significant, *p < 0.05, **p < 0.01. NBF: non-biofilm forming, WBF: weak biofilm forming, SBF: strong biofilm forming, MBF: moderate biofilm forming. The code numbers refer to the isolate numbers for raw milk (RM), and kareish cheese (KCh) samples.

**Figure 10:**
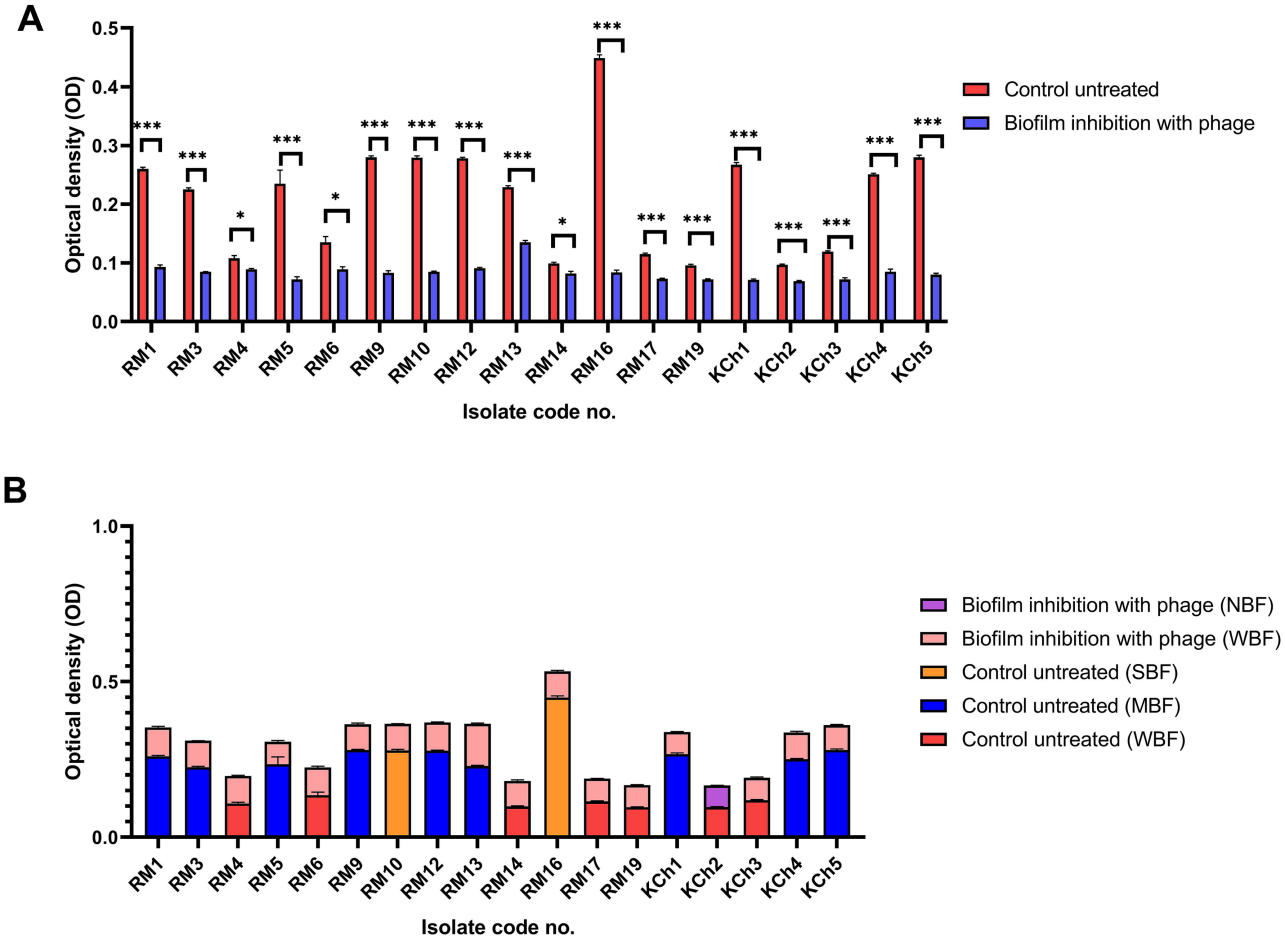
Antibiofilm effect of vB_LmoP_M15 phage on the inhibition of *Listeria monocytogenes* biofilms **(A)**. Differences in biofilm production abilities among control untreated *L. monocytogenes* isolates and vB_LmoP_M15 phage treated isolates at the initiation of biofilms formation **(B)**. ns: non-significant, *p < 0.05, ***p < 0.0001. NBF: non-biofilm forming, WBF: weak biofilm forming, SBF: strong biofilm forming, MBF: moderate biofilm forming. The code numbers refer to the isolate numbers for raw milk (RM), and kareish cheese (KCh) samples.

The vB_LmoP_M15 phage demonstrated a strong antibiofilm activity. The reduction in biofilm biomass was consistent across both preformed biofilms and inhibition of biofilm formation, highlighting the phage’s potential as an effective antibiofilm agent (Supplementary Table 3; Figures 8–10). There were statistically significant differences (*p* < 0.05) in biofilm formation between all control untreated biofilms and phage-treated biofilms at both the preformed biofilm and inhibition of biofilm formation (Supplementary Table 3). These findings underscore the phage’s ability to inhibit biofilm formation and disrupt established biofilms, making it a promising candidate for controlling *L. monocytogenes*.

#### 3.12.1 Disruption of preformed biofilm

The efficacy of vB_LmoP_M15 in inhibiting preformed biofilms was evaluated by treating 48-h-old biofilms with a phage solution (10^9^ PFU/ml). A notable decrease in biofilm biomass was recorded in the wells treated with phage compared to the untreated control wells. The OD values of the phage-treated biofilms were markedly lower, demonstrating the phage’s ability to disrupt the established biofilms (*p* < 0.05 to *p* < 0.0001) (Figure 9A). The most notable reduction was observed in isolate RM16, where the OD decreased from 0.449 ± 0.0052 to 0.077 ± 0.002 (phage-treated) (*p* < 0.0001) (Figure 9A). Two SBF isolates (RM10 and RM16) were reduced to MBF and WBF, respectively, after phage treatment at the preformed biofilm stage (Figure 9B).

#### 3.12.2 Inhibition of biofilm formation

The potential of vB_LmoP_M15 to prevent biofilm formation was investigated by co-incubating *L. monocytogenes* with phage solution (10^9^ PFU/ml) at the initiation of biofilm formation. After 48 h of incubation, a significant decrease in biofilm formation was observed in phage-treated wells compared to the control wells. The OD values of phage-treated wells were significantly lower, confirming the phage’s ability to inhibit biofilm formation (*p* < 0.05 to *p* < 0.0001) (Figure 10A). Two SBF isolates (RM10 and RM16) were reduced to WBF after phage treatment and one WBF isolate become NBF (Figure 10B).

### 3.13 Lytic phage reduces *L. monocytogenes* counts in pasteurized milk

The efficacy of vB_LmoP_M15 for inhibition of *L. monocytogenes* (KCh5) growth in pasteurized milk was evaluated. Pasteurized milk samples, which were initially inoculated with *L. monocytogenes* (3.0 log10 CFU/ml), underwent treatment with vB_LmoP_M15 phage at a Multiplicity of Infection (MOI) of 100 and were incubated at 30°C for a duration of 7 days.

**Figure 11:**
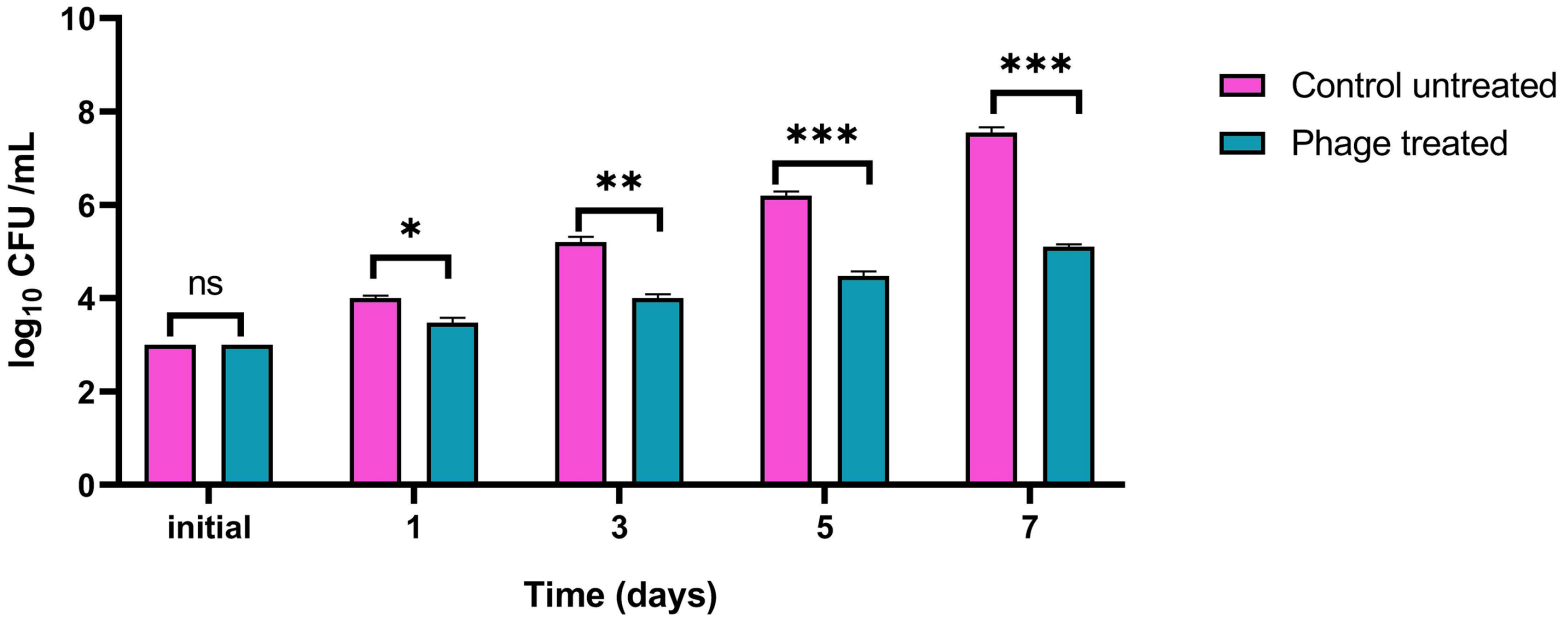
Inhibition of *Listeria monocytogenes* growth by vB_LmoP_M15 phage in pasteurized milk at 30°C. vB_LmoP_M15 phage was applied at an MOI of 100. Initial inoculum size of the host bacteria in milk media was 3.0 log10 CFU/ml. Asterisks denote statistically significant differences between phage-treated and control groups at each time point: ns, not significant; *p < 0.05, **p < 0.01, ***p < 0.001.

In the untreated control samples, *L. monocytogenes* exhibited substantial growth, with counts increasing from the initial 3.0 log_10_ CFU/ml to 7.55 log_10_ CFU/ml by day 7 (Figure 11). Conversely, treatment with vB_LmoP_M15 phage significantly inhibited the growth of *L. monocytogenes*. While bacterial counts in the phage-treated group increased to 5.1 log_10_ CFU/ml by day 7, this represented a marked attenuation of growth compared to the control.

A significant reduction in *L. monocytogenes* counts was observed in the phage-treated milk relative to the untreated control milk starting from day 1. Specifically, a log_10_ reduction of 0.523 (*p* = 0.011) was recorded by day 1. This inhibitory effect became more pronounced over the incubation period, with log_10_ reductions reaching 1.200 (*p* = 0.001) by day 3, 1.723 (*p* < 0.0001) by day 5, and culminating in a maximum observed log10 reduction of 2.45 (*p* < 0.0001) by day 7 (Figure 11).

## 4 Discussion

This study aimed to address the significant public health challenge posed by *L. monocytogenes* in the Egyptian dairy sector by investigating its antimicrobial resistance patterns, biofilm formation ability, and the potential of bacteriophage biocontrol, thereby providing crucial insights for enhancing food safety. A notable prevalence of *Listeria* spp. (20%) was found, with *L. monocytogenes* specifically detected in 15% of the total samples. Significantly, raw milk exhibited a high contamination rate (31.7% for *Listeria* spp., with 25% being *L. monocytogenes*), while Kareish cheese showed a lower but still concerning prevalence (8.3% for *Listeria* spp., with 5% being *L. monocytogenes*). These findings, align with the global understanding of raw dairy products as significant vehicles for *Listeria* transmission (Gandhi and Chikindas, 2007; Jackson et al., 2018). However, the prevalence noted in raw milk in this study seems significantly greater than the reported averages in various other regions. For instance, meta-analyses and reviews suggest average global prevalence rates in raw milk often range from approximately 3 to 7% (e.g., a recent meta-analysis reported 3.44% (Li et al., 2024), while older reviews cited ranges like 3–7% in North America (Bangieva and Rusev, 2017)). Similarly, although the prevalence of *L. monocytogenes* in soft cheeses varies considerably, the 8.3% detected in Kareish cheese aligns with globally reported ranges. Nonetheless, some reviews suggest that the average positivity rates for certain types of soft cheeses typically fall between 4 and 5% (Knight et al., 2008). The elevated prevalence in raw milk reflects specific local factors, including potential gaps in farm hygiene, milking practices, lack of widespread pasteurization adoption, storage conditions, and the traditional methods often used in Kareish cheese production, compounded by the widespread consumption of raw milk in certain communities (Jackson et al., 2018). The detection of *L. welshimeri* in raw milk further suggests environmental contamination routes, possibly from soil or water sources, consistent with observations in agricultural settings (Vázquez-Boland et al., 2001).

Compounding the issue of prevalence, the AMR profiles observed in the *Listeria* isolates from this study present a significant public health concern. Notably, the high resistance rates to certain antibiotics commonly used in veterinary and human medicine in Egypt stand out. For instance, the universal resistance (100%) to the third-generation cephalosporin cefotetan and high resistance to fosfomycin (91.7%), amoxycillin (83.3%), and clindamycin (79.2%) observed across isolates are alarming. Comparing these findings internationally, particularly with European data, reveals potential disparities. While AMR in *L. monocytogenes* is a growing global issue, some recent European surveillance studies suggest that acquired resistance rates, especially to clinically crucial antibiotics like ampicillin, remain relatively low in isolates from both food and clinical sources there (Moura et al., 2024). Studies from France, for example, have previously reported varying resistance levels, but the extremely high rates for agents like cefotetan and fosfomycin seen here may be less common in European food isolates (Morvan et al., 2010). This disparity likely underscores the consequences of factors potentially more prevalent in the Egyptian context, such as the less regulated use of antibiotics in the livestock sector, which can drive the selection and spread of resistant strains (World Health Organization, 2021). The identification of a PDR *L. monocytogenes* isolate in Kareish cheese, is a particularly critical finding, as such strains are rarely reported in foodborne *Listeria* globally and represent a severe therapeutic challenge. However, it is also important to note the observed susceptibility of all isolates in this study to vancomycin, teicoplanin, gentamicin, and levofloxacin, suggesting these agents may remain viable treatment options, although continued surveillance is crucial.

The ability of *L. monocytogenes* to form biofilms further exacerbates the challenges associated with its control and potential resistance. Biofilm formation is a critical factor contributing to its persistence in food processing environments and its resistance to sanitizers and stress conditions (Djordjevic et al., 2002). This study investigated the ability of *L. monocytogenes* isolates from raw milk and Kareish cheese to form biofilms. A significant proportion of isolates demonstrated the ability to form biofilms, with the majority classified as weak (WBF) or moderate (MBF) biofilm formers (38.46% WBF, 46.15% MBF in raw milk; 40% WBF, 60% MBF in Kareish cheese). Notably, strong biofilm formers were detected among raw milk isolates (15.38%). While no statistically significant difference was found between the sources (*p* = 0.68), the presence of biofilm-forming strains, including strong formers, in the dairy chain is concerning. Biofilms act as protective niches, potentially enhancing bacterial survival during processing and storage, and critically, they are known to contribute significantly to increased resistance to antimicrobials and disinfectants. Bacteria within biofilms can exhibit resistance levels orders of magnitude higher than their planktonic counterparts due to a combination of factors. These include the physical barrier presented by the extracellular polymeric substance matrix, which can limit antibiotic penetration; altered metabolic states and slower growth rates of bacteria deep within the biofilm, making them less susceptible to certain antibiotics; the presence of persister cells, which are phenotypically tolerant to high antibiotic concentrations; and the potential for increased horizontal gene transfer of resistance determinants within the dense biofilm community (Liu et al., 2024). The identification of these biofilm-forming isolates, including MDR and PDR strains (Figures 4A,B), underscores the complex interplay between persistence mechanisms and resistance, highlighting the dual challenge *L. monocytogenes* poses to food safety in Egypt and the critical importance of control strategies that can effectively target biofilms.

Considering these challenges, exploring alternative control strategies like bacteriophage therapy is essential. This study focused on isolating and characterizing a novel lytic bacteriophage, vB_LmoP_M15, sourced from an Egyptian dairy farm environment, which may serve as a potential biocontrol agent against *L. monocytogenes*. *Listeria*-specific bacteriophages exhibit considerable diversity, primarily belonging to the *Siphoviridae* and *Myoviridae* families. However, members of the *Podoviridae* family have also been identified and applied in *listerial* control, such as the phage vB_LmoP_M15 isolated in the present study (Sulakvelidze et al., 2001; Klumpp and Loessner, 2013). The morphological characterization of vB_LmoP_M15 via TEM revealed an icosahedral head and a short non-contractile tail, consistent with the *Podoviridae* family. Such phages are often strictly lytic, making them suitable candidates for biocontrol as they effectively kill host bacteria without the risk of integrating into the bacterial genome or transferring undesirable genes (Sulakvelidze et al., 2001; Hagens and Loessner, 2014). The restriction enzyme analysis revealed clear digestion patterns, and the genome size of vB_LmoP_M15 was estimated to be around 48.5 kb, suggesting genetic stability and helping to distinguish it from other phages. In addition, the one-step growth curve showed a short latent period (15–20 min) and a high burst size (172 PFU per infected cell), reflecting efficient and rapid phage replication. Our characterization showed vB_LmoP_M15 possesses moderate thermal stability (up to 50°C) and optimal stability at neutral pH, though it retains activity in mildly acidic conditions (pH 4–5). These characteristics are crucial for potential application in food environments, which can vary in temperature and pH. While sensitive to prolonged UV exposure, its stability profile suggests viability in various dairy processing and storage scenarios. Crucially, vB_LmoP_M15 demonstrated significant lytic activity against MDR and PDR *L. monocytogenes* isolates. Furthermore, its pronounced antibiofilm activity, effectively disrupting preformed biofilms and inhibiting biofilm formation, is particularly significant. Biofilms represent a major challenge in *L. monocytogenes* control, and phages capable of disrupting these structures are highly sought after (Djordjevic et al., 2002; Grigore-Gurgu et al., 2024). Importantly, the efficacy of vB_LmoP_M15 in pasteurized milk was confirmed, demonstrating significant reduction of *L. monocytogenes* counts compared to controls, achieving a maximum reduction of 2.45 log10 (*p* < 0.0001) by day 7 (Figure 11). The application of bacteriophages as biocontrol agents in food, particularly in dairy products, is gaining considerable interest globally. Recent studies continue to demonstrate their potential for controlling various pathogens; for instance, phages from the Myoviridae family have been characterized for reducing *Staphylococcus aureus* growth and biofilms in milk (Gharieb et al., 2020), while others, particularly from the *Herelleviridae* family, have shown efficacy against *Listeria monocytogenes* in milk (Stone et al., 2020; Şanlıbaba and Buzrul, 2022). Although challenges related to the food matrix exist, several phage-based products are already approved for use against *L. monocytogenes* on ready-to-eat foods in some countries (Khan et al., 2023). The isolation and characterization of vB_LmoP_M15, effective against MDR and PDR strains and their biofilms, represents a significant step forward in developing targeted, natural intervention strategies to enhance the safety of dairy products. Although genome annotation provided promising preliminary insights, we acknowledge that whole genome sequencing (WGS) was not conducted in the present study. This is an important limitation, and we recommend WGS as a necessary next step to confirm the absence of lysogenic elements, antimicrobial resistance genes, or virulence factors.

## 5 Conclusion

This study underscores the considerable challenge presented by *L. monocytogenes* in raw milk and Kareish cheese, marked by elevated prevalence rates and concerning levels of antimicrobial resistance, including PDR strain. Furthermore, the ability of these isolates to form biofilms complicates control efforts, potentially contributing to both environmental persistence and reduced susceptibility to treatments. However, the isolation and characterization of the novel lytic bacteriophage vB_LmoP_M15 offers a promising avenue for biocontrol. This phage demonstrated potent activity against the circulating resistant *L. monocytogenes* strains and crucially exhibited significant efficacy in both preventing biofilm formation and disrupting established biofilms. These findings underscore the potential of vB_LmoP_M15 phage as a targeted, natural strategy to mitigate *L. monocytogenes* contamination and enhance the safety of dairy products, particularly in the face of increasing antimicrobial resistance.

## Data Availability

The original contributions presented in the study are included in the article/Supplementary material, further inquiries can be directed to the corresponding author.

## Footnotes

^1^ https://www.r-project.org/
