# Supplementary figures and images for: Lytic bacteriophage disrupts biofilm and inhibits growth of pan-drug-resistant Listeria monocytogenes in dairy products
Source: Front Microbiol. 2025 Aug 4;16:1653368. doi: 10.3389/fmicb.2025.1653368 (PMC12358432; doi:10.3389/fmicb.2025.1653368)

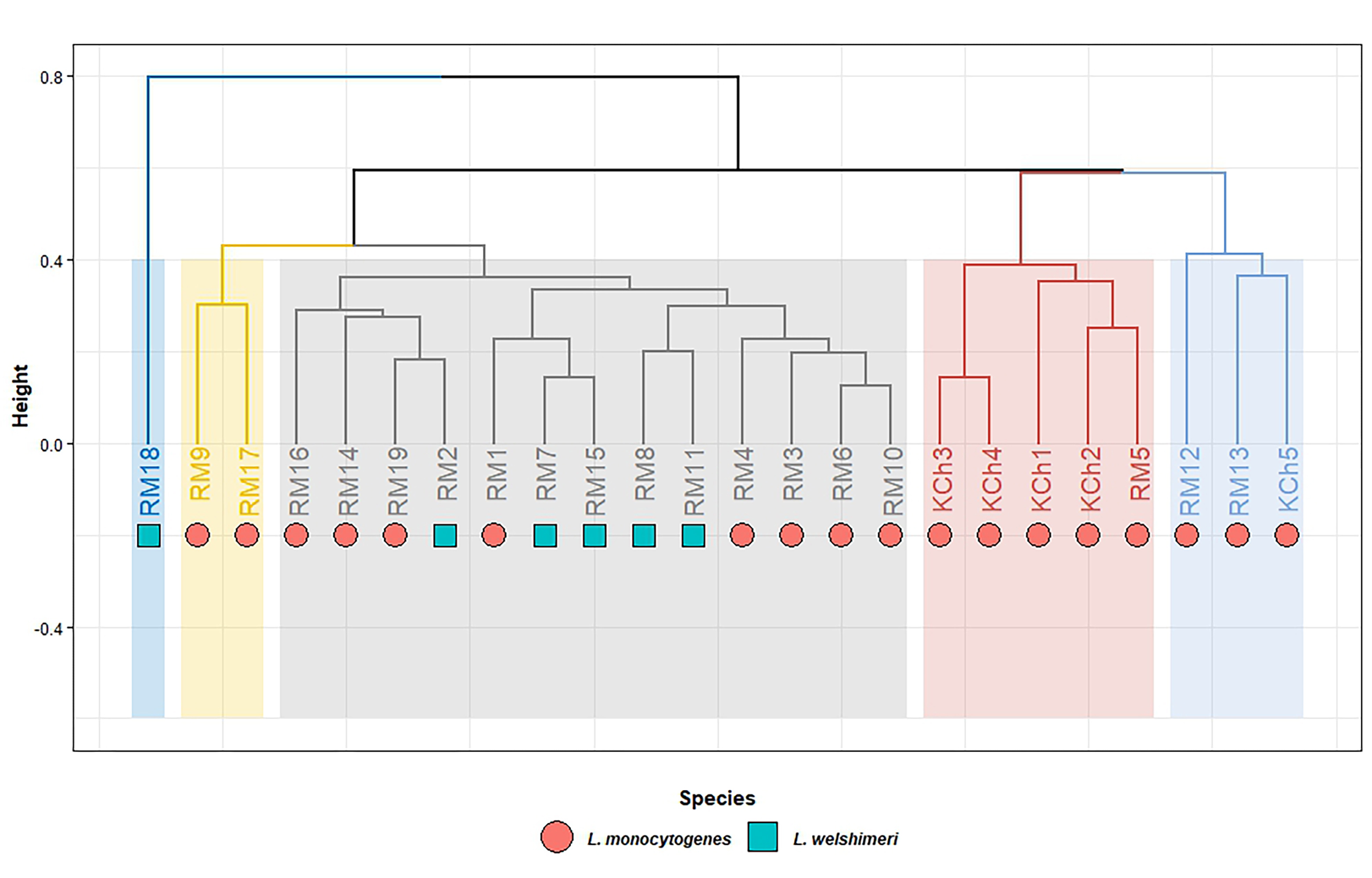

Supplement: Supplementary file 1 [file Image_1.jpeg]

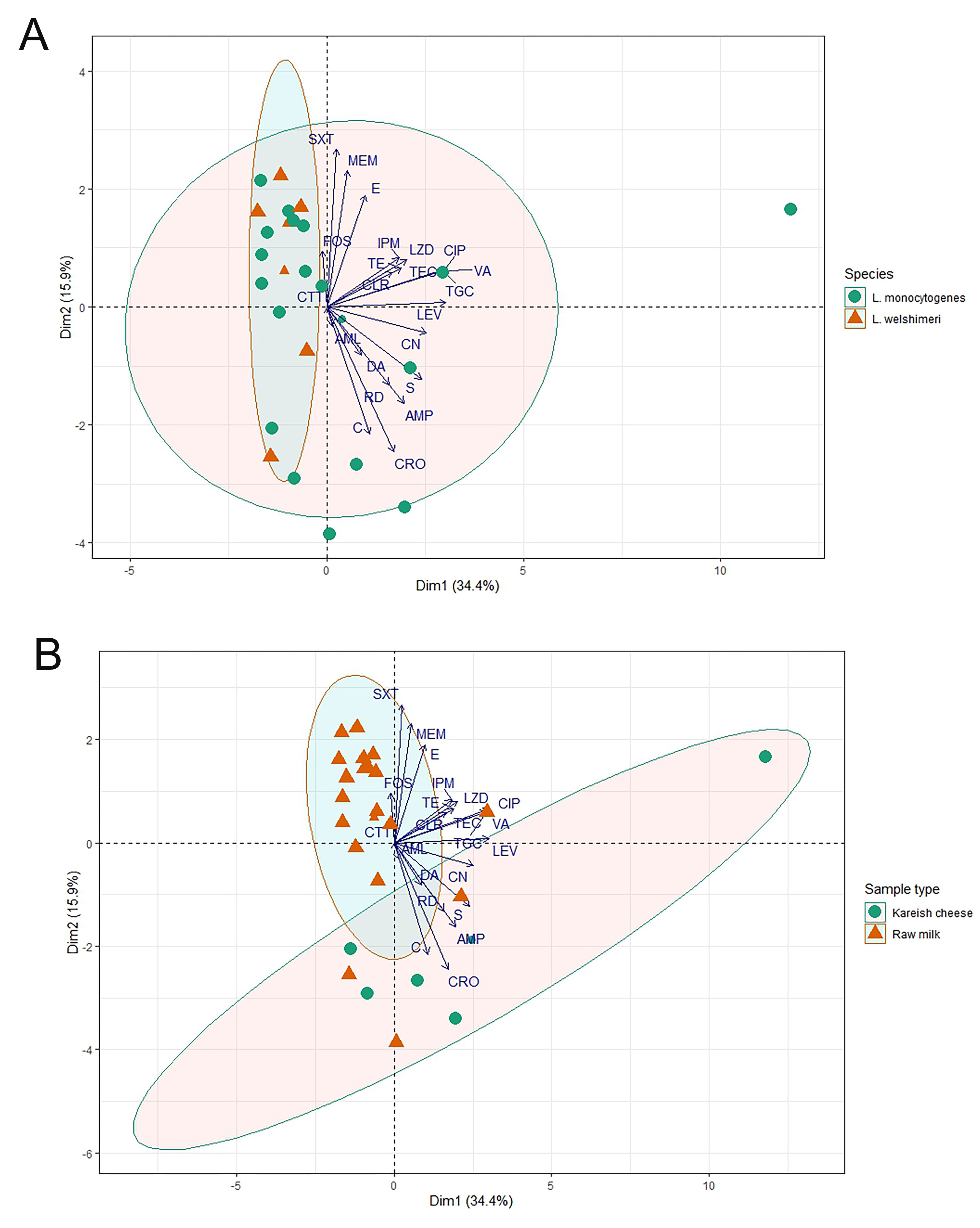

Supplement: Supplementary file 2 [file Image_2.jpeg]

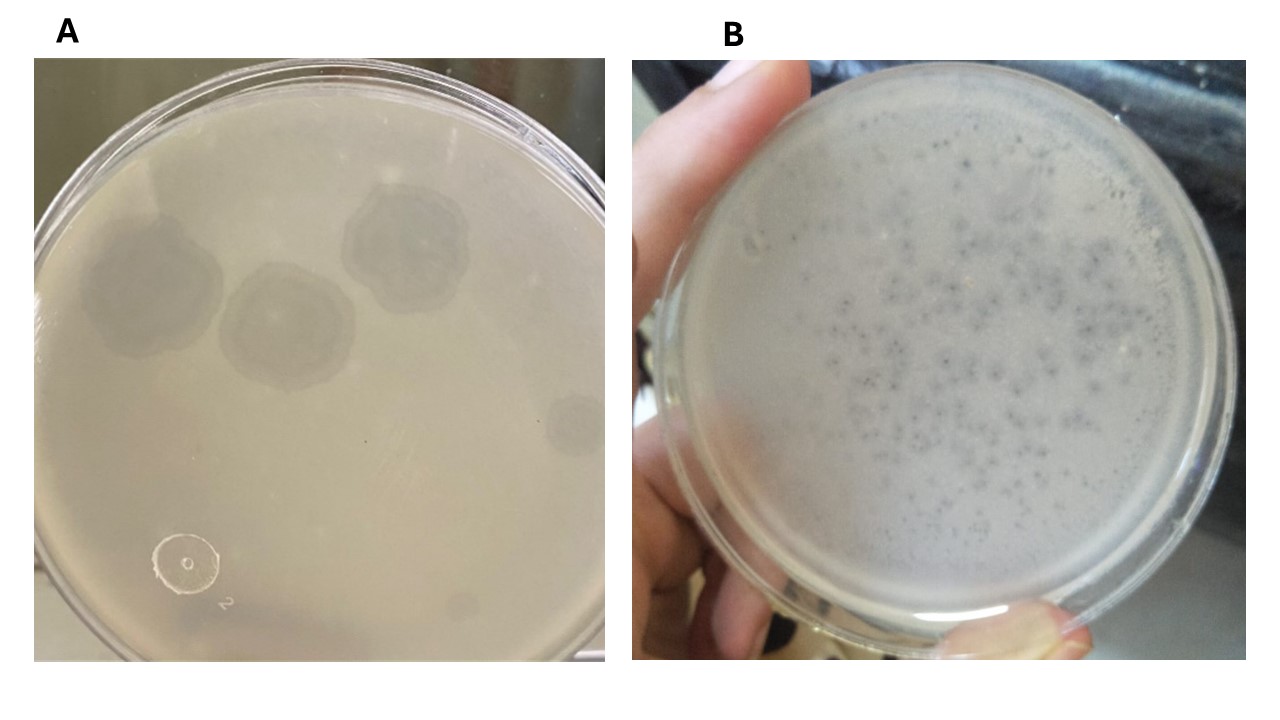

Supplement: Supplementary file 3 [file Image_3.jpeg]

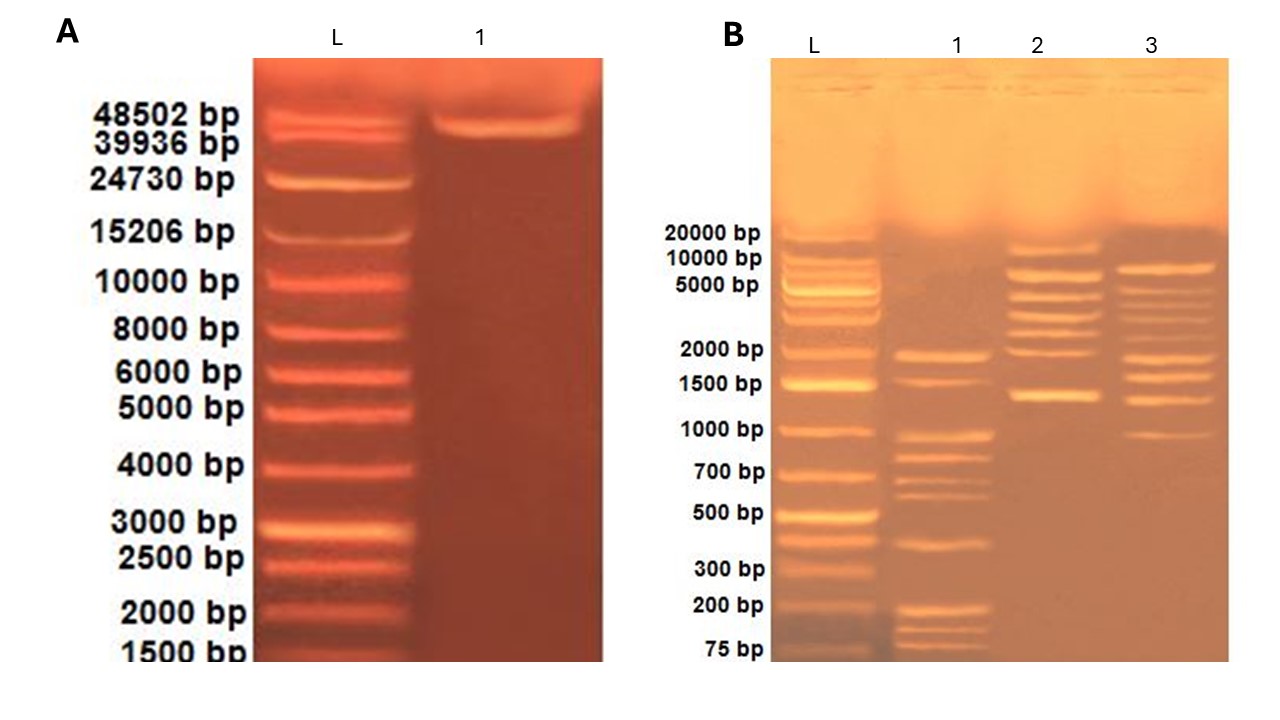

Supplement: Supplementary file 4 [file Image_4.jpeg]
