# Supplementary material for: Lytic bacteriophage disrupts biofilm and inhibits growth of pan-drug-resistant Listeria monocytogenes in dairy products
Source: Front Microbiol. 2025 Aug 4;16:1653368. doi: 10.3389/fmicb.2025.1653368 (PMC12358432; doi:10.3389/fmicb.2025.1653368)
Supplement: Supplementary file 5 [file Table_1.docx]

Supplementary Material

**Supplementary Table 1.** Antimicrobial resistance rates of *Listeria* species isolates

| **Antimicrobial class/ subclass** | | **AMA** | **Conc. (μg)** | **No. of Listeria isolates (%)** | | ***P*-value** | **Total no. of isolates**  **(n=24)** |
| --- | --- | --- | --- | --- | --- | --- | --- |
|  |  |  |  | **Raw milk (n= 19)** | **Kareish cheese (n= 5)** |  |  |
| Glycopeptides | Glycopeptide | VA | 30 | 0 | 1 (20) | 0.208 | 1 (4.17) |
|  | Lipoglycopeptides | TEC | 30 | 0 | 1 (20) | 0.208 | 1 (4.17) |
| β-Lactams | Penems/Carbapenems | MEM | 10 | 16 (84.21) | 2 (40) | 0.078 | 18 (75) |
|  |  | IPM | 10 | 3 (15.79) | 1 (20) | 1 | 4 (16.67) |
|  | Cephems/ Cephalosporins III | CRO | 30 | 5 (26.32) | 4 (80) | 0.047 | 9 (37.5) |
|  | Cephems/ Cephamycins | CTT | 30 | 19 (100) | 5 (100) | NA | 24 (100) |
|  | Penicillins | AML | 10 | 15 (78.95) | 5 (100) | 0.544 | 20 (83.33) |
|  |  | AMP | 10 | 1 (5.26) | 3 (60) | 0.018* | 4 (16.67) |
| Aminoglycosides | | CN | 10 | 2 (10.53) | 2 (40) | 0.179 | 4 (16.67) |
|  |  | S | 10 | 2 (10.53) | 3 (60) | 0.042* | 5 (20.83) |
| Fluoroquinolones | | LEV | 5 | 0 | 1 (20) | 0.208 | 1 (4.17) |
|  |  | CIP | 5 | 0 | 1 (20) | 0.208 | 1 (4.17) |
| Macrolides | | E | 15 | 8 (42.11) | 2 (40) | 1 | 10 (41.67) |
|  |  | CLR | 15 | 3 (15.79) | 2 (40) | 0.544 | 5 (20.83) |
| Phenicols | | C | 30 | 6 (31.58) | 5 (100) | 0.011* | 11 (45.83) |
| Folate pathway antagonists | | SXT | 25 | 15 (78.95) | 1 (20) | 0.028* | 16 (66.67) |
| Fosfomycins | | FOS | 200 | 17 (89.47) | 5 (100) | 1 | 22 (91.67) |
| Lincosamides | | DA | 2 | 14 (73.68) | 5 (100) | 0.316 | 19 (79.16) |
| Tetracyclins |  | TE | 30 | 2 (10.53) | 1 (20) | 1 | 3 (12.5) |
|  | Glycylcyclines | TGC | 15 | 0 | 1 (20) | 0.208 | 1 (4.17) |
| Oxazolidinones | | LZD | 30 | 1 (5.26) | 1 (20) | 0.38 | 2 (8.3) |
| Ansamycins | Rifamycins | RD | 5 | 7 (36.84) | 3 (60) | 0.615 | 10 (41.67) |

VA: vancomycin, TEC: teicoplanin, MEM: meropenem, IPM: imipenem, CRO: ceftriaxone, CTT: cefotetan, AML: amoxycillin, AMP: ampicillin, CN: gentamicin, S: streptomycin, LEV: levofloxacin, CIP: ciprofloxacin, E: erythromycin, CLR: clarithromycin, C: chloramphenicol, SXT: trimethoprim/sulfamethoxazole (cotrimoxazole), FOS: fosfomycin, DA: clindamycin, TE: tetracycline, TGC: tigecycline, LZD: linezolid, RD: rifampicin. NA: non-applicable; * *p* >0.05.

**Supplementary Table 2.** Host range of lytic *L. monocytogenes* phage against different bacterial strains isolated from milk.

| **Tested bacteria** | **Lytic area** | |
| --- | --- | --- |
|  | **Spot test** | **Titer of plaque PFU/mL** |
| *Pseudomonas aeruginosa* (*P. aeruginosa*) M1  *P. aeruginosa* M2  *P. aeruginosa* M3 | -  -  - | 0  0  0 |
| *Staphylococcus aureus* (*S. aureus*) M4  *S. aureus* M5  *S. aureus* M6 | +++  ++  +++ | 2.2 x10^7^  5.0 x10^6^  6.0 x10^8^ |
| *Escherichia coli* (*E. coli*) M7  *E. coli* M8  *E. coli* M9 | -  +  - | 0  1.5 x10^6^  0 |
| *Listeria monocytogenes* (*L. monocytogene*) KCh5  *L. monocytogene* RM19  *L. monocytogene* RM12 | +++  ++  +++ | 9.2 x10^8^  9 x10^6^  1.2 x10^7^ |
| *Streptococcus agalactiae* M10  *S. agalactiae* M11  *S. agalactiae* M12 | +  +  + | 2.7 x10^6^  9.0 x10^5^  3.3x10^6^ |

+: positive, -: negative.

**Supplementary Table 3.** Antibiofilm effect of vB_LmoP_M15 phage on *Listeria monocytogenes* preformed biofilm and inhibition of biofilm formation

| **Isolate Code no.** | **Untreated biofilm** | | **Category of biofilm** | **Eradication of preformed biofilm** | | **Category of biofilm** | **Inhibition of biofilm formation** | | **Category of biofilm** | ***p*-value** |
| --- | --- | --- | --- | --- | --- | --- | --- | --- | --- | --- |
|  | OD | SEM |  | OD | SEM |  | OD | SEM |  |  |
| RM1 | 0.26 | 0.0028 | MBF | 0.098 | 0.002 | WBF | 0.093 | 0.0035 | WBF | <0.0001*** |
| RM3 | 0.225 | 0.0028 | MBF | 0.097 | 0.002 | WBF | 0.085 | 3.00E-04 | WBF | <0.0001*** |
| RM4 | 0.108 | 0.0046 | WBF | 0.088 | 0.005 | WBF | 0.089 | 0.0017 | WBF | 0.025* |
| RM5 | 0.235 | 0.023 | MBF | 0.099 | 0.008 | WBF | 0.072 | 0.0043 | WBF | <0.0001*** |
| RM6 | 0.135 | 0.0098 | WBF | 0.091 | 0.0098 | WBF | 0.089 | 0.0044 | WBF | 0.003** |
| RM9 | 0.28 | 0.0023 | MBF | 0.166 | 0.005 | MBF | 0.083 | 0.0037 | WBF | <0.0001*** |
| RM10 | 0.279 | 0.0034 | SBF | 0.197 | 0.022 | MBF | 0.085 | 0.0011 | WBF | <0.0001*** |
| RM12 | 0.278 | 0.0017 | MBF | 0.102 | 3.00E-04 | WBF | 0.091 | 0.0012 | WBF | <0.0001*** |
| RM13 | 0.229 | 0.0023 | MBF | 0.173 | 0.006 | MBF | 0.135 | 0.0032 | WBF | <0.0001*** |
| RM14 | 0.099 | 0.0023 | WBF | 0.087 | 0.0009 | WBF | 0.082 | 0.0036 | WBF | 0.008** |
| RM16 | 0.449 | 0.0052 | SBF | 0.077 | 0.002 | WBF | 0.084 | 0.0036 | WBF | <0.0001*** |
| RM17 | 0.115 | 0.0017 | WBF | 0.089 | 0.003 | WBF | 0.073 | 0.0008 | WBF | <0.0001*** |
| RM19 | 0.096 | 0.0017 | WBF | 0.083 | 0.003 | WBF | 0.072 | 0.001 | WBF | <0.0001*** |
| KCh1 | 0.267 | 0.004 | MBF | 0.204 | 0.013 | MBF | 0.071 | 0.0017 | WBF | <0.0001*** |
| KCh2 | 0.097 | 0.0011 | WBF | 0.077 | 0.002 | WBF | 0.069 | 0.001 | NBF | <0.0001*** |
| KCh3 | 0.119 | 0.0017 | WBF | 0.097 | 0.004 | WBF | 0.072 | 0.0026 | WBF | <0.0001*** |
| KCh4 | 0.251 | 0.0017 | MBF | 0.137 | 0.012 | WBF | 0.085 | 0.0044 | WBF | <0.0001*** |
| KCh5 | 0.28 | 0.0034 | MBF | 0.077 | 0.001 | WBF | 0.08 | 0.0026 | WBF | <0.0001*** |

NBF: non-biofilm forming, WBF: weak biofilm forming, SBF: strong biofilm forming, MBF: medium biofilm forming. The code numbers refer to the isolate numbers for raw milk (RM), and kareish cheese (KCh) samples. **p*< 0.05, ****p*< 0.0001.

**Supplementary Figure 1.** Hierarchical clustering dendrogram shows the relatedness of *Listeria* species isolates as determined by the antimicrobial resistance profiles.

**Supplementary Figure 2.** Principal components analysis (PCA) biplot of antimicrobial resistance profiles of *Listeria* isolates from different species (A), and sources (B).

Supplementary Figure 3. Lytic activity of bacteriophage vB_LmoP_M15 against *L.monocytogenes* isolates. Spot test demonstrating lysis zones formed by vB_LmoP_M15 on lawns of *L. monocytogenes* isolate RM19 (A). (B) Plaque morphology of vB_LmoP_M15 formed on a lawn of *L. monocytogenes* isolate, displaying distinct clear plaques.

**Supplementary Figure 4.** Genomic DNA analysis of bacteriophage vB_LmoP_M15. (A) Agarose gel electrophoresis (0.6%) of extracted vB_LmoP_M15 genomic DNA. Lane L, DNA ladder; Lane 1, Undigested vB_LmoP_M15 genomic DNA. (B) Restriction fragment length polymorphism (RFLP) analysis of vB_LmoP_M15 genomic DNA resolved on agarose gel. Lane L, DNA ladder; Lane 1: vB_LmoP_M15 DNA digested with *Hinf*I; Lane 2: vB_LmoP_M15 DNA digested with *Hind*III; and Lane 3: vB_LmoP_M15 DNA digested with *Hae*III.
